# Statistical Learning Methods for Neuroimaging Data Analysis with Applications

**DOI:** 10.1146/annurev-biodatasci-020722-100353

**Published:** 2023-04-26

**Authors:** Hongtu Zhu, Tengfei Li, Bingxin Zhao

**Affiliations:** 1 Department of Biostatistics, Department of Statistics, Department of Genetics, and Department of Computer Science, University of North Carolina, Chapel Hill, North Carolina, USA; 2 Biomedical Research Imaging Center, University of North Carolina, Chapel Hill, North Carolina, USA; 3 Department of Radiology, University of North Carolina, Chapel Hill, North Carolina, USA; 4 Department of Statistics and Data Science, University of Pennsylvania, Philadelphia, Pennsylvania, USA

**Keywords:** causal pathway, heterogeneity, image processing analysis, neuroimaging techniques, population-based statistical analysis, study design

## Abstract

The aim of this review is to provide a comprehensive survey of statistical challenges in neuroimaging data analysis, from neuroimaging techniques to large-scale neuroimaging studies and statistical learning methods. We briefly review eight popular neuroimaging techniques and their potential applications in neuroscience research and clinical translation. We delineate four themes of neuroimaging data and review major image processing analysis methods for processing neuroimaging data at the individual level. We briefly review four large-scale neuroimaging-related studies and a consortium on imaging genomics and discuss four themes of neuroimaging data analysis at the population level. We review nine major population-based statistical analysis methods and their associated statistical challenges and present recent progress in statistical methodology to address these challenges.

## 1. INTRODUCTION

Neuroimaging refers to the process of producing images of the structure, function, or pharmacology of the central nervous system (CNS). It has been a dynamic and evolving field with (*a*) the development of new acquisition techniques, (*b*) the collection of various neuroimaging data in clinical settings and medical research, and (*c*) the development of statistical learning (SL) methods. Popular neuroimaging techniques include structural magnetic resonance imaging (sMRI), functional magnetic resonance imaging (fMRI), diffusion weighted imaging (DWI), computerized tomography (CT), positron emission tomography (PET), electroencephalography (EEG), magnetoencephalography (MEG), and functional near-infrared spectroscopy (fNIRS). These techniques were developed to measure specific tracers in CNS that are directly and indirectly associated with brain structure and function. For instance, PET delineates how an injected radioactive tracer (e.g., fluorodeoxyglucose) moves and accumulates in the brain, whereas fMRI measures an indirect tracer, called the concentration of deoxyhemoglobin, in the flow downstream of the activated neurons caused by the brain’s activity. The development of SL methods for individual neuroimaging data raises serious challenges for existing SL methods due to four themes: (T1) complex brain objects, (T2) complex spatiotemporal structures, (T3) extremely high dimensionality, and (T4) heterogeneity within subjects and across groups.

**Figure 1:**
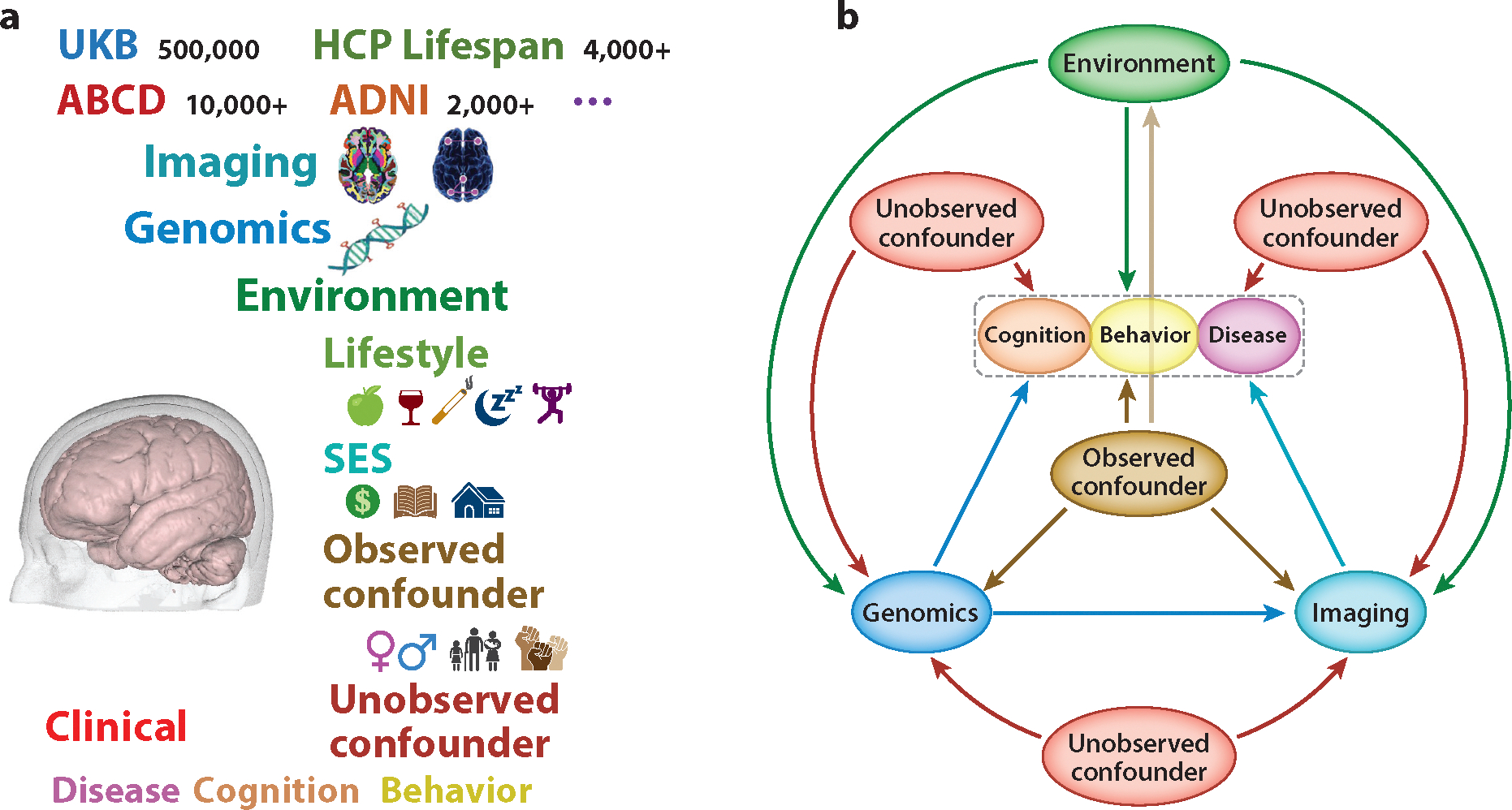
(*a*) Major data types from different domains in several representative large-scale biomedical studies. The number after each dataset represents the sample size. (*b*) A dynamic causal model for delineating the CGIC pathway confounded with environmental factors and unobserved confounders. An arrow from a factor X to a factor Y represents the direct effect of X on Y. Abbreviations: ABCD, Adolescent Brain Cognitive Development; ADNI, Alzheimer’s Disease Neuroimaging Initiative; CGIC, causal genetic-imaging-clinical; HCP, Human Connectome Project; SES, socioeconomic status; UKB, UK Biobank.

In recent years, huge amounts of neuroimaging data have been collected in healthcare, biomedical research studies, and clinical trials. Neuroimaging has the potential to improve clinical care for diagnosis and prognosis in various brain-related diseases, such as dementia, sleep disorders, and schizophrenia. Some typical uses of neuroimaging include identifying the effects of brain-related diseases (e.g., stroke or glioblastoma), locating cysts and tumors, and finding swelling and bleeding. Many large-scale biomedical studies have collected or are collecting massive amounts of neuroimaging data (e.g., sMRI, DWI, and fMRI) with high spatial and/or temporal resolution as well as other complex information (e.g., genomics and health factors) to map the human brain connectome in order to better understand the pathophysiology of brain-related disorders, the progress of neuropsychiatric and neurodegenerative disorders, normal brain development, and the diagnosis of brain cancer, among other things. In the last two decades, there have been at least three pioneering neuroimaging-related studies, including the Alzheimer’s Disease Neuroimaging Initiative (ADNI) (http://www.adni-info.org/) (1), the Human Connectome Project (HCP) (http://humanconnectome.org/consortia/) (2), and the UK Biobank (UKB) study (https://www.ukbiobank.ac.uk/) (3). These represent major advances and innovations in acquisition protocols, analysis pipelines, data management, experimental design, and sample size. Figure 1a shows multiview data across different domains (e.g., imaging, genetics, or environmental factors) in some large-scale biomedical studies. Neuroimaging biomarkers have many uses in clinical trials for drug development in neurological and psychiatric disorders (4). These uses include providing tools for screening trial participants, establishing biodistribution, assaying target engagement, and measuring pharmacodynamic activity, as well as monitoring safety and providing an evidence measure of disease modification. The development of SL methods for clinical translation and large-scale neuroimaging-related studies raises serious challenges to existing statistical methods due to four additional themes: (T5) sampling bias, (T6) complex missing patterns, (T7) complex data objects, and (T8) complicated causal pathways in brain disorders.

There is a large literature on the development of SL methods for neuroimaging data analysis (NDA) that correlate multiple types of data from different domains across multiple studies, eventually establishing a dynamic causal pathway, such as the causal genetic-imaging-clinical (CGIC) pathway, as shown in Figure 1b, that links genetics to brain (or neuroimaging) phenotypes and clinical outcomes confounded with health factors. These SL methods can be categorized into two categories: image processing analysis (IPA) at the individual level and population-based statistical analysis (PSA) for samples of subjects. We further group IPA methods into deconvolution and structure learning methods (5–10). Deconvolution methods primarily include image reconstruction and image enhancement. Structure learning methods primarily include image segmentation and image registration. Due to themes T1–T4 and the lack of high-quality annotated datasets, it is very challenging to develop good IPA pipelines to extract a relatively small number of image phenotypes with high repeatability and reproducibility for both individual healthcare and PSA. We group the various PSA methods into nine main categories, including study design, statistical parametric mapping (SPM), object-oriented data (OOD) analysis, dimensional reduction (DR) methods, data integration (DI), imputation methods, predictive models, imaging genetics, and causal discovery (11–16). Due to themes T1–T8, each category has its own statistical challenges, requiring specific statistical methodologies to address them. However, the development of scalable PSA methods has fallen seriously behind the technological advances in neuroimaging techniques, making it difficult to translate research findings into clinical practice.

## 2. NEUROIMAGING TECHNIQUES AND IMAGE PROCESSING ANALYSIS METHODS

**Figure 2:**
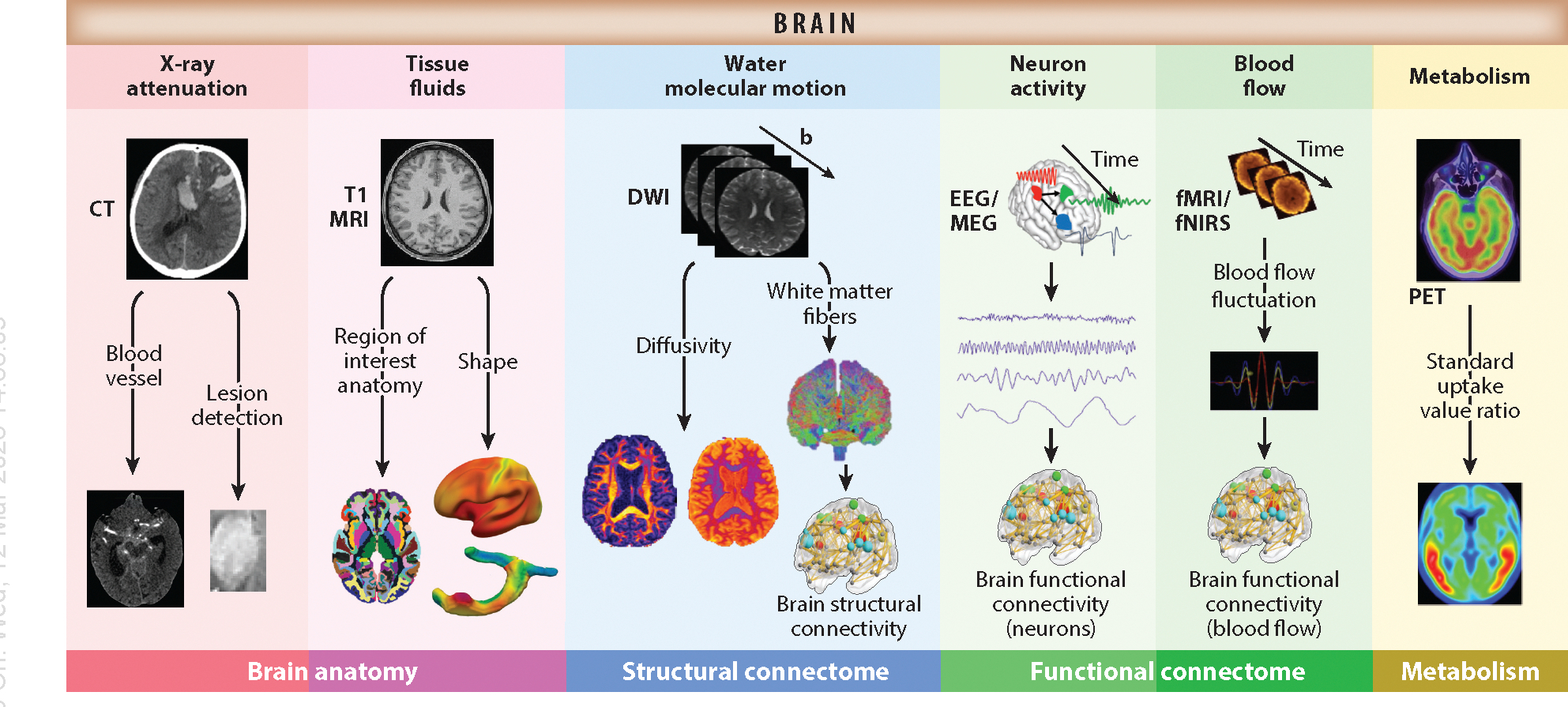
Roles of different imaging modalities for extracting various types of features. Abbreviations: **b**, *b*-vector parameter; CT, computerized tomography; DWI, diffusion weighted imaging; EEG, electroencephalography; (f )MRI, (functional) magnetic resonance imaging; fNIRS, functional near-infrared spectroscopy; MEG, magnetoencephalography; PET, positron emission tomography.

We briefly review eight neuroimaging techniques below. For each image modality, its tracer, data dimension, features, main uses, and key software (17) are also described in Supplemental Table 1 and illustrated in Figure 2. There is great interest in developing integration methods to fuse together neuroimaging data from different modalities (18) since no single modality can completely capture the complex dynamics of brain physiology and pathology. This allows us to synthesize complementary information from different modalities, leading to a comprehensive picture of the brain under different clinical conditions, tasks, and resting states, as well as under normal development. The three categories of multimodal neuroimaging are structural–structural combinations, functional–functional combinations, and structural–functional combinations. fMRI/EEG DI, which is an example of a functional–functional combination, improves both the spatial and temporal resolution of data while cross-validating findings across different scales. Simultaneous CT-MRI scanners, which are examples of structural–structural combinations, integrate the high-contrast resolution of MRI with the high spatial resolution of CT. Structural–functional combinations, such as EEG/sMRI, PET/CT, and PET/MRI, link anatomical structure data with functional dynamics data, improving the mapping of brain anatomy to brain functions and the simulation of brain dynamics. Furthermore, scientists have proposed whole-brain models by combining anatomical networks extracted from DWI/sMRI with local dynamics extracted from fMRI/EEG/MEG and metabolism extracted from PET (19). These whole-brain models usually consist of three basic elements: brain parcellation [e.g., multimodal parcellation (MMP) from the HCP (20)], an anatomical connectivity matrix for the human connectome, and local dynamics for the activity of each brain region and interaction terms with other regions.

In the following subsections, we discuss four themes of neuroimaging data, review existing major IPA methods for processing neuroimaging data, and delineate major statistical challenges associated with IPA.

### 2.1. Themes T1–T4

We discuss four themes of neuroimaging data as follows.

#### 2.1.1. T1: complex brain objects.

All neuroimaging modalities are developed to indirectly (or directly) measure the structure and function of the cerebrum, cerebellum, brain stem, diencephalon (thalamus and hypothalamus), limbic system, reticular activating system, and ventricular system in the human brain. For instance, the cerebrum is part of the forebrain, consisting of the cerebral cortex of gray matter in the outer layer and white matter (WM) in the inner layer. It is responsible for language processing, motor function, memory, vision, personality, and other cognitive functions. The cerebral cortex consists of the frontal lobe, temporal lobe, parietal lobe, and occipital lobe, while its surface is made up of gyri and sulci. Moreover, the human brain uses neurons as information messengers to send electrical impulses and chemical signals to different regions of the brain and body in order to control biological functions and react to environmental changes. Moreover, there are two sets of blood vessels, the vertebral arteries and the carotid arteries, that supply blood and oxygen to the brain. These objects in the brain are the targets of different neuroimaging modalities.

#### 2.1.2. T2: complex spatiotemporal structures.

There are three spatiotemporal aspects of the neuroimaging datasets, including spatiotemporal resolutions, spatiotemporal smoothness, and spatiotemporal correlation. In Supplemental Table 1 we show different resolution ranges for the eight neuroimaging techniques. In general, higher spatial (or temporal) resolution leads to better spatial (or temporal) localization, but in some cases (e.g., DWI), higher spatial resolution decreases the signal-to-noise ratio. Due to the intrinsic smooth structure of different brain regions discussed in theme T1, neuroimaging data are expected to contain spatially contiguous regions or effect regions with relatively sharp edges, showing locally strong spatiotemporal smoothness and spatiotemporal correlation. Moreover, long-range temporal correlations among different brain regions may be caused by respiration, cardiac rhythm, and cognitive processes.

#### 2.1.3. T3: extremely high dimensionality.

Both raw neuroimaging data and extracted feature data can be extremely high dimensional even for a single subject. For instance, for a single subject, the number of 3D DWI images, each of which consists of over 500,000 voxels, varies from dozen to a few hundred, and the extracted feature data include 3D images of various diffusion-related quantities [e.g., diffusion tensors and fractional anisotropy (FA)], a whole-brain tractographic dataset (which can contain more than 1,000,000 streamlines), diffusion properties along WM bundles, and structural connectivity network metrics. For a single subject, the number of 3D task-based fMRIs is about several hundred, and the extracted feature data include 3D activation patterns, region-based activation and interaction patterns, and weighted and binary network metrics.

#### 2.1.4. T4: heterogeneity within individual subjects and across centers/studies.

Neuroimaging data may be written as

**Figure 3:**
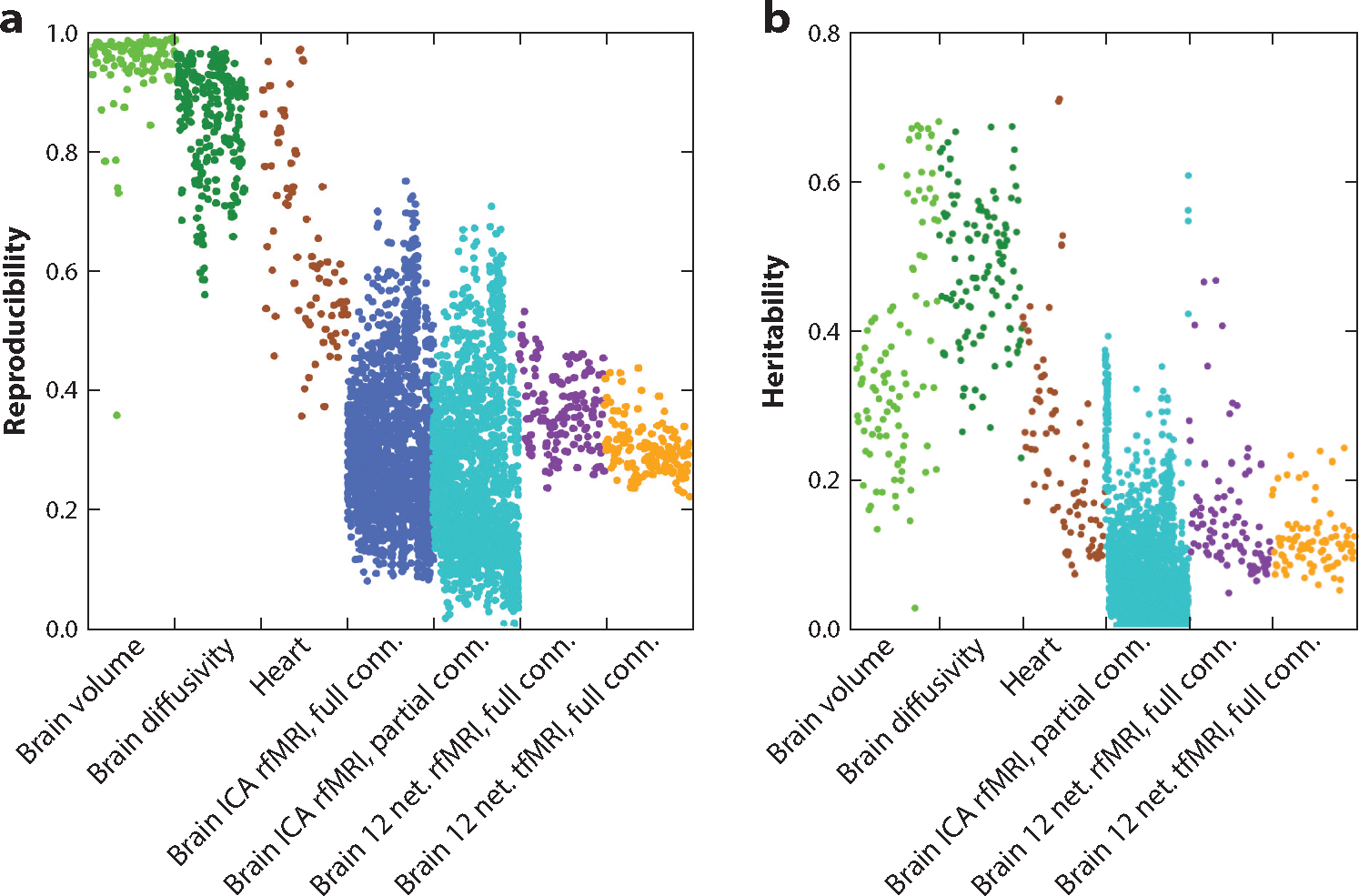
The reproducibility (*a*) and heritability (*b*) of seven categories of imaging traits based on UK Biobank data, including brain regional volume, brain diffusivity parameters, heart MRI traits, brain ICA-based rfMRI full and partial connectivity, 12-region network–based brain rfMRI full connectivity, and 12-region network–based brain tfMRI full connectivity. Abbreviations: 12 net., 12-region network; conn., connectivity; fMRI, functional MRI; ICA, independent component analysis; MRI, magnetic resonance imaging; rfMRI, resting state fMRI; tfMRI, task-based fMRI.

1. I=f(brain(age,gene,race,disease,otherfactors),device,acquisitionparams.,noise),

where noises contain all kinds of noise components (e.g., thermal noise or motion) (14) and brain includes both structural and functional components. Equation 1 emphasizes two important facts: (*a*) that neuroimaging data represent a mixture of different components introduced by the brain, the imaging device, the acquisition parameters, and different noises, and (*b*) that brain changes may be caused by age, genes, race, disease, and other factors (e.g., stimulus, lifestyle, or environmental factors). The effect of device, acquisition parameters, and noises in I can be larger than the effect of brain changes caused by predictors of interest. For a single subject in a short time window, it is expected that structural images are much more stable than functional images even in the same scanner, whereas one may observe visible differences in the same type of structural images acquired using two different scanners. A sensible neuroimaging modality requires that brain changes caused by a specific condition are large relative to the variability caused by noises, acquisition parameters, and devices. Figure 3a presents the reproducibility using intraclass correlation coefficient values of imaging phenotypes based on the UKB test–retest dataset. We observe that the brain and heart structural traits have much larger reproducibility than the brain functional traits, suggesting the complexity and variability of brain function.

Any novel IPA methods for neuroimaging data need to account for some or all of the challenges connected to the four themes T1–T4 discussed above. Below we review two categories of IPA methods, including deconvolution and structure learning, in the existing literature.

### 2.2. Image Processing Analysis: Deconvolution

We use the term “deconvolution” to represent all computational and statistical methods for reconstructing image data of interest from recorded imaging signals with various noise components. We can further categorize all deconvolution methods into the image reconstruction and enhancement processes (9, 21).

The image reconstruction process for neuroimage data aims to reconstruct clinically interpretable images from raw data acquired by neuroimaging devices. For instance, MRI data are acquired in k-space and a specific image reconstruction process is needed to generate MRI images in image space. Several key methods for MRI reconstruction include noise prewhitening, zero filling in k-space, raw data filtering, Fourier transforms, and phased array coil combination (21). Recently, compressed sensing algorithms and deep learning (DL) methods have played a critical role in fast MRI acquisition and reconstruction (22, 23). Furthermore, most neuroimage data in the image space still need additional reconstruction in order to estimate local features of interest in the human brain. Some examples include diffusion tensors for DWI, cortical surface for sMRI, WM fiber bundles for DWI, and hemodynamic response functions for fMRI and fNIRS (11, 24–28).

The image enhancement process for neuroimaging data improves the quality of generated images for better presentation and analysis. Popular enhancement tasks include denoising, superresolution, bias field correction, and harmonization. Among them, bias field correction and harmonization were proposed to correct for two major confounders, including devices and artifacts in noises, as described in Equation 1. Specifically, bias field in image data is the presence of a low-frequency intensity nonuniformity, representing a potential confounder in various image analysis tasks, such as tissue segmentation (29). Various bias correction methods (e.g., the non-parametric nonuniform intensity normalization algorithm) can be divided into prospective and retrospective approaches according to the different sources of bias field and the different features used in bias correction (29). Harmonization in imaging data aims to correct significant inter- and intrasite variability even within individual subjects, which may be caused by hardware, reconstruction processes, and acquisition parameters. Such variability is much more profound across subjects in multisite and multistudy neuroimaging datasets. Therefore, there is a great interest in the development of various harmonization methods for correcting inter- and intrasite variability in neuroimaging datasets, including the surrogate variable approach, meta-analysis, mega-analysis, the removal of an artificial voxel effect by linear regression, phantome-based harmonization, DL, or ComBat (combined association test) (30, 31). Readers are referred to Section 3.2.5 for further details.

### 2.3. Image Processing Analysis: Structure Learning

We use the term “structure learning” to refer to all computational and statistical methods for extracting signals of interest from reconstructed imaging data. We can further categorize structure learning methods into the image segmentation and registration processes (5–8, 32–36).

The image segmentation process for neuroimage data aims to label reconstructed neuroimaging data into meaningful subgroups for clinical and scientific tasks, including the quantification of brain development, the localization of pathology, surgical planning, and image-guided interventions. Existing image segmentation methods can be roughly clustered into traditional segmentation techniques (e.g., intensity-based methods or surface-based methods), machine learning approaches, and DL approaches, such as fully connected networks and U-nets (35, 37). Major neuroimage segmentation tasks include skull stripping, cortical and subcortical structures segmentation, WM tract parcellation, functional parcellation, and lesion localization (37–42). Performing these tasks allows researchers to extract a wealth of important features while addressing themes T1–T4, including local properties of brain structures; short-, median-, and long-range structural and functional connectivity patterns; and structural and functional markers.

Segmentation tasks have at least three important applications. First, they greatly compress the dimensionality of neuroimaging data, as detailed in theme T3, while providing strong biological interpretation. Second, refined brain structural and functional parcellations greatly improve our understanding of the organizational principles behind the human brain across multiple regions, multiple scales, and multiple tasks. Third, an important clinical application of image segmentation is computer-aided detection and diagnosis for localizing lesions and then classifying them into a specific lesion type (7).

The image registration process for neuroimaging data aims to transform the spatial coordinates of neuroimaging data within individual subjects or across different subjects into the same coordinate system of an atlas (32–36). Some important applications of registration include the construction of brain atlases, multimodal fusion, the quantification of brain development, population analysis, longitudinal analysis, automated image segmentation, shape analysis, and the localization of pathology. Most image registration algorithms have three major components including (*a*) the similarity measure, (*b*) the transformation model, and (*c*) the optimization process. The similarity measures can be either intensity based (e.g., mutual information or correlation metrics) or feature based (e.g., distances between image features such as points, lines, and contours). The transformation models can be categorized into rigid (translations and rotations), affine, homography, and deformation. Deformation models (5) can be further grouped into physics-based, interpolation-based, and knowledge-based approaches, leading to ill-posed problems. Such models usually require imposing implicit and explicit regularization constraints, such as hard constraints, topology preservation, volume preservation, and rigidity constraints. Recently, there has been a growing interest in DL-based image registration methods, such as deep iterative registration, deep supervised registration, and deep unsupervised registration (32). These DL-based models hold great promise for completing registration within a few seconds using a single forward calculation, with an accuracy comparable to conventional methods.

As an example, we consider the construction of imaging-based human brain atlases as one of the most important applications of registration. Cartographic approaches have been widely used to create anatomical atlases (e.g., Brodmann’s map and Dejerine’s map) based on postmortem tissues, establishing spatial correspondences between a coordinate and a brain structure. Recently, there has been a tremendous evolution of human brain atlases (e.g., Yeo-Network, Atlas of the Human Brain in Stereotaxic Space, or HCP-MMP) (20, 39, 43, 44) due to the availability of many advanced imaging techniques, brain mapping methods, large-scale neuroimaging datasets, and registration methods, among others. Various criteria have been used for human brain atlases, including brain architecture, functional activity, anatomical and functional connectivity, abnormality, genetic and protein information, cell type, lifespan, spatiotemporal scale, ethnicity, and multiple modalities, among others. In the near future, modern human brain atlases may provide an integrative and comprehensive description of brain structure and function in large populations and across scales, ages, genders, behavioral tasks, ethnic groups, disease states, and imaging modalities.

### 2.4. A Generic Statistical Model for Image Processing Analysis

Here we discuss a generic statistical model for IPA, including denoising, superresolution, reconstruction, segmentation, and registration. First, we consider image reconstruction. Suppose that we observe $\left\{\left(x_{i},I_{i}\right):i=1,\ldots ,n\right\}$, where $I_{i}$ and $x_{i}$ are, respectively, an imaging vector and a predictor vector, which may depend on the imaging device, acquisition parameters, and observable confounders in noise components. It is assumed that $I_{i}$ given $x_{i}$ follows a probability distribution $p\left(I_{i}|h\left(x_{i},\theta \right),\sigma \right)$, where θ is a vector of parameters (or functions), σ is a vector of nuisance parameters, and h(⋅,⋅) is a vector of functions. Let us now consider two examples. First, we consider the raw sMRI data in k-space. In this case, $I_{i}$ is the complex MRI measurement in k-space, $x_{i}$ includes its ($k_{x},k_{y}$) coordinate and other MRI scanner parameters, n is the total number of observations in k-space, and θ is the sMRI in image space. Second, we consider the DWI data. In this case, $I_{i}$ is the DWI image, $x_{i}$ includes b-values and diffusion directions, n is the total number of DWI volumes, and θ is the image of diffusion tensors.

The primary interest of many deconvolution methods is to estimate θ by maximizing

2. $$L_{n}(\theta )=\sum_{i=1}^{n}log\;p\left(I_{i}|h\left(x_{i},\theta \right),\sigma \right)+R_{1}\left(h\left(x_{i},\theta \right)\right)+R_{2}(\theta ,\sigma ),$$

where $R_{1}(\cdot )$ and $R_{2}(\cdot )$ are two regularization terms based on prior information, such as sparsity and spatiotemporal structures in T1 and T2. As an illustration, we discuss how to construct $log\;p\left(I_{i}|h\left(x_{i},\theta \right),\sigma \right)$ in Equation 2 for image denoising by using weighted loss functions. Many denoising methods solve a weighted loss function by incorporating signals in the neighboring locations of each location. A further refinement is to build a sequence of increasing neighborhood sizes and then sequentially fit the weighted loss function in Equation 2 to estimate θ as size increases from the smallest size to the largest size, while borrowing information from the previous sizes (45, 46). In this case, $L_{n}(\theta )$ may implicitly depend on all observations in the neighboring locations of each location, so it is strongly dependent on both location and neighborhood size. Specifically, we estimate θ in Equation 2 at the smallest size, denoted as ${\overset{ˆ}{\theta }}_{\left(0\right)}$, and then use adaptive smoothing methods to sequentially calculate ${\overset{ˆ}{\theta }}_{\left(s_{k}\right)}$ for $s_{0}=0<s_{1}<\cdots <s_{K}$, while preserving spatial smoothness and edges (47).

Both image segmentation and registration can be also formulated as special cases of Equation 2. For image segmentation, $x_{i}$ and $I_{i}$ are, respectively, input image data for segmentation and output segmentation results, n is the number of annotated images, and $R_{1}(\cdot )$ may be a spatiotemporal regularization term. For image registration, we consider registering a pair of images, with $x_{i}$ and $I_{i}$ being source image and target image, respectively. In this case, n=1, $h\left(x_{i},\theta \right)=x_{i}\left(T_{i}(s)\right)$ with $T_{i}(\cdot )$ being a transformation model, $logp\left(I_{i}|h\left(x_{i},\theta \right),\sigma \right)$ is a matching criterion chosen to match $\left(I_{i},x_{i}\right)$, and $R_{1}\left(x_{i}\left(T_{i}(s)\right)\right)$ is imposed on $T_{i}(\cdot )$ to induce certain constraints (e.g., diffeomorphism) (32–36).

### 2.5. Challenges

We have briefly reviewed four major IPA techniques including reconstruction, enhancement, segmentation, and registration, which are the key building blocks of most neuroimaging preprocessing pipelines, but each requires substantial efforts at validation, which can be a daunting task. For instance, most neuroimaging segmentation methods suffer from a major data bottleneck (or barrier) for validation even though the segmentation accuracy of DL-based methods has significantly outperformed traditional methods. Specifically, there is no single, publicly available, high-quality neuroimaging dataset with detailed annotation information that covers a large spectrum of segmentation tasks in neuroimaging research, which greatly limits the translation of segmentation methods to the clinic. In contrast, publicly available datasets and environments (e.g., ImageNet) played a vital role in the development of DL methods for computer vision problems and in the successes of narrow artificial intelligence systems, such as DeepMind’s AlphaGo. Several methodological attempts to partially address the data bottleneck for validation include unsupervised learning, self-supervised learning (SSL), weakly supervised learning, data augmentation, patchwise training, and transfer learning (7, 35, 37). However, several key developments are greatly needed in order to address the data bottleneck, including the development of good annotation protocols for major segmentation tasks; the collection of high-quality datasets covering a wide range of settings, as discussed in theme T4; the use of active learning and reinforcement learning (48, 49); and a comprehensive evaluation system for image segmentation and registration. Similar comments also apply for validating most image registration methods.

As an illustration, we consider a comprehensive DWI preprocessing pipeline consisting of (*a*) fiber orientation reconstruction, (*b*) WM tracking, (*c*) WM parcellation, (*d*) WM registration, (*e*) extraction of diffusion properties along WM and structural connectivity metrics, (*f*) visualization, and (*g*) statistical analysis. Although major technical advancements have been made in these steps in the last decade, steps *b*–*d* still face major technical barriers. Specifically, multiple tractography challenges reveal that most state-of-the-art algorithms produce many more false WM bundles than valid ones (50, 51), leading to erroneous structural connectivity metrics. Those false WM bundles are mainly caused by the limitation of DWI and the complexity of WM structure, as discussed in theme T1. Moreover, a recent open call for segmenting 14 WM fascicles based on the same sets of streamlines obtained from six subjects (52) reveals that there is large variability across 57 different state-of-the-art segmentation protocols and techniques for such a task. This variability is mainly caused by the complexity of WM structure, as discussed in theme T1, and the lack of good validation datasets, in addition to the limitations of existing clustering techniques. The variability in WM tracking and parcellation greatly affects metrics for downstream extraction and quantification of WM connectivity (52). Another technical barrier is that existing WM registration algorithms not only suffer from pinching effects for transforming WM bundles to the WM bundle atlas (36) but also largely ignore the diffusion property information along fiber tracts (53), causing a local misalignment issue among those diffusion property functions. In contrast, the method of tract-based spatial statistics (TBSS) (54), which projects WM diffusion properties onto a whole-brain WM skeleton, is a robust approach with high reproducibility (Figure 3), but TBSS does not have individual fiber tract specificity.

## 3. POPULATION-BASED STATISTICAL ANALYSIS METHODS

**Figure 4:**
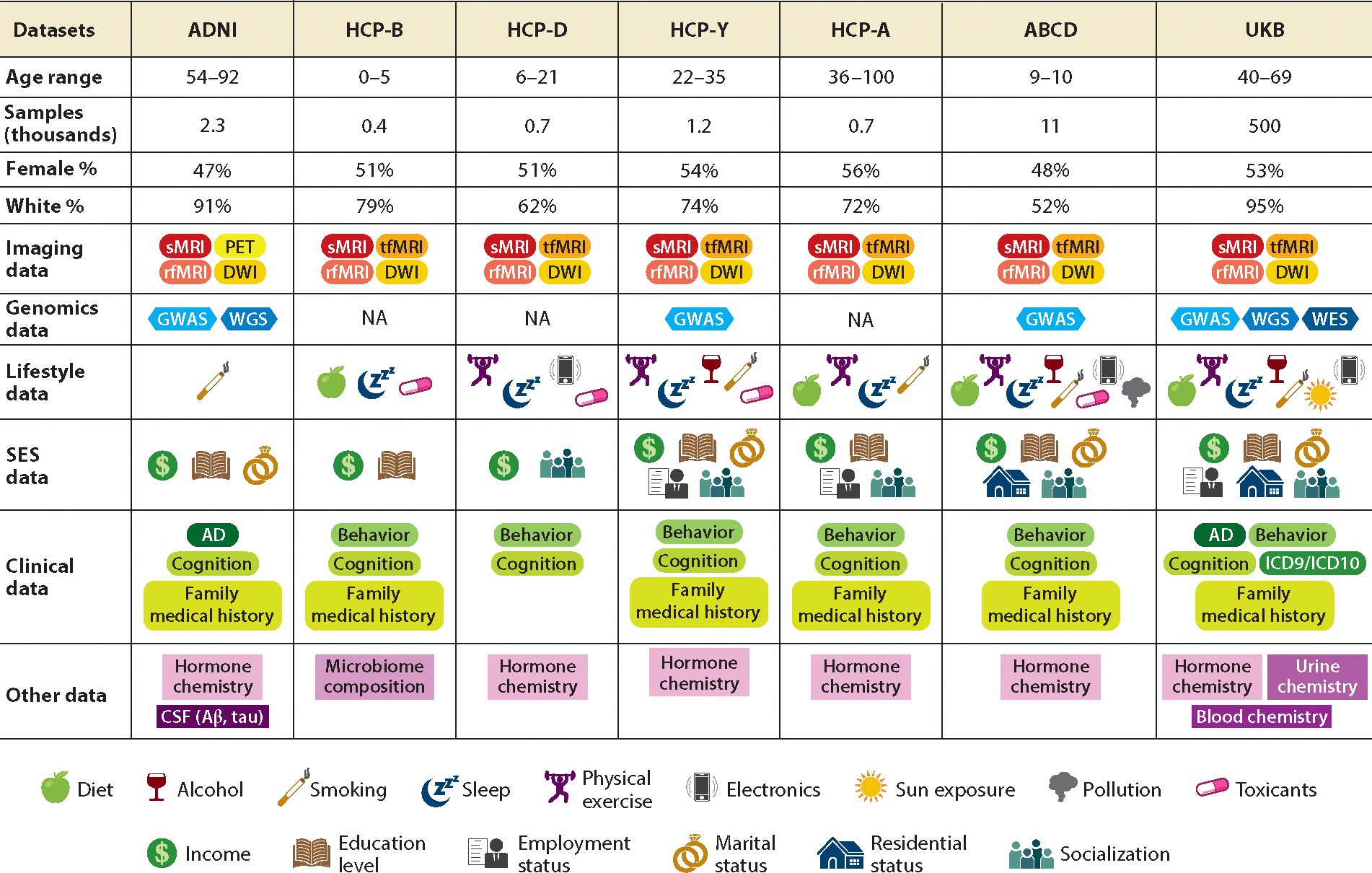
Some summary information for datasets from the ADNI, HCP, ABCD and UKB studies (until 2021). Abbreviations: Aβ, amyloid-beta; ABCD, Adolescent Brain Cognitive Development; AD, Alzheimer’s disease status; ADNI, Alzheimer’s Disease Neuroimaging Initiative; CSF, cerebrospinal fluid; DWI, diffusion weighted imaging; GWAS, genome-wide association study; HCP, Human Connectome Project; ICD9/ICD10, International Classification of Diseases, 9th/10th Edition; MRI, magnetic resonance imaging; NA, not any; PET, positron emission tomography; rfMRI, resting state functional MRI; SES, socioeconomic status; sMRI, structural MRI; tfMRI, task-based functional MRI; UKB, UK Biobank; WES, whole-exome sequencing; WGS, whole-genome sequencing.

Over the past decade, we have witnessed an exponential increase in neuroimaging data collected in many large-scale biomedical studies (e.g., UKB) primarily due to huge investments from various funding agencies and the private sector (3, 55). The number of subjects in a neuroimaging study has increased from several dozen in most neuroimaging-related studies 30 years ago to more than 10,000 in several studies more recently. Besides neuroimaging data, these large-scale biomedical studies are collecting other data types, including genetic data, behavioral data, environmental factors, and clinical outcomes, in order to better understand the progress of, for example, neuropsychiatric disorders, neurological disorders, stroke, and normal brain development. Recently, several large consortia have been formed to enhance collaborations on neuroimaging and imaging genomics among researchers across the world. In the Supplemental Appendix we discuss four large-scale neuroimaging-related studies and the imaging genomics ENIGMA (Enhancing NeuroImaging Genetics through Meta Analysis) Consortium, whose detailed information is also included in Figure 4.

In the following sections we discuss four themes of NDA in large-scale biomedical studies, review existing major PSA methods for NDA, and discuss major statistical challenges associated with PSA.

### 3.1. Themes T5–T8

Although we have already discussed the themes T1–T4 of neuroimaging data, four more themes arise from the joint analysis of big neuroimaging data and other related variables in many large-scale biomedical studies, such as UKB and ENIGMA.

#### 3.1.1. T5: sampling bias.

The most important issue in NDA is how to appropriately address potential sampling bias introduced at design and data collection stages. Some common types of sampling bias include undercoverage, observer bias, voluntary response bias, survivorship bias, recall bias, and exclusion bias (56). A direct consequence of sampling bias is that the sample in a study is not a representative sample of a target population. Sampling bias can have profound effects on downstream data analysis, as well as on the generalizability and fairness (e.g., sex, race, or age) of conclusions drawn from statistical models. Although the issue of sampling bias is prevalent in neuroimaging research, it has been largely ignored in the medical imaging literature until recently (57, 58). Appropriately dealing with sampling bias requires specific strategies in the study design and data collection stages, as well as explicit statistical models of the sample selection process (59).

#### 3.1.2. T6: complex missing patterns.

Missing data are frequently encountered in large-scale neuroimaging studies for various reasons, such as the data not being included in the study design, faulty scanning, attrition in longitudinal studies, data misentry, and nonresponses in surveys. For a given variable that has missing data, there are three types of missingness: missing at random (MAR), missing completely at random (MCAR), and missing not at random (MNAR). Simply ignoring missing observations and improperly imputing them may lead to efficiency loss and introduce spurious correlations. Additional challenges arise in handling missing data in large-scale neuroimaging-related studies. For instance, variables with different missing patterns often occur in the same neuroimaging study, while high-dimensional image data are blockwise missing either within individual studies or across different studies. Little progress has been made on how to appropriately integrate information across domains from heterogeneous studies in the presence of blockwise missing data (60) even though there is a large literature on handling missing entries for low-dimensional clinical outcomes (61, 62).

#### 3.1.3. T7: complex data objects.

Complex data objects in curved spaces frequently arise in the process of extracting biologically meaningful features from neuroimaging data. Some examples of data objects include planar shapes, symmetric positive definite (SPD) matrices, matrix Lie groups, tree-structured data, the Grassmann manifolds, deformation fields, connectivity graphs, functional connectivity graphs, diffusivity properties along WM bundles, and the shape representations of cortical and subcortical structures, among others. Most of these complex data objects are inherently nonlinear and high dimensional (or even infinite dimensional), so many traditional statistical techniques, including semiparametric and nonparametric regression, growth curve models, clustering, classification, correlation, and DR, are often not directly applicable (36, 63–68). The efficient analysis of complex data objects and variables obtained from other domains presents major statistical and computational challenges.

#### 3.1.4. T8: complicated causal pathways in brain disorders.

Brain disorders such as Alzheimer’s disease (AD) affect one in six people worldwide, posing a great threat to public health and resulting in significant disability, morbidity, and mortality. Most approved therapies for brain disorders only treat symptoms. Existing studies suggest that most complex brain disorders are highly heritable with polygenic architecture and are caused by a combination of genetic and clinical risk factors (3, 69–71). Moreover, many brain disorders can be regarded as endpoints of abnormal trajectories of brain changes. Since neuroimaging data are closer representations of the underlying biology and can be measured temporally, much effort has been devoted to understanding the temporal CGIC pathophysiological pathway in the continuum of brain disease progression from increasingly large cohorts (e.g., ADNI). These efforts may lead to the identification of possibly hundreds of risk genes and clinical factors that contribute to abnormal developmental trajectories of brain disorders. Once such an identification has been accomplished, we may establish a set of complex causal relationships that delineate the CGIC pathways confounded by environmental factors and unobserved confounders, as shown in Figure 1. These risk factors can be detected early enough to identify therapies urgently needed to correct abnormal developmental trajectories, ultimately preventing the onset of brain disorders or reducing their severity.

### 3.2. Population-Based Statistical Analysis Methods

There is great interest in developing SL methods for NDA in order to address issues arising from themes T1–T4 inherent in neuroimaging data, discussed in Section 2.1, and themes T5–T8 inherent in large-scale neuroimaging studies, discussed in Section 3.1. Here we briefly review nine categories of PSA methods in the literature, many of which are emerging. Moreover, there are many important papers in each category that we cannot cite due to space limitations.

#### 3.2.1. Study designs.

Popular designs in large-scale observational studies include case–control, cross-sectional, and cohort studies (56, 59). These designs can be applied to a variety of scientific questions, but they all have certain limitations when it comes to specific clinical and epidemiological applications. Case–control studies are good for studying rare clinical outcomes and latent diseases. Participants in a case–control study are selected based on their outcome status and are defined as cases and controls. In such studies, matching is often used to ensure that the cases and controls have similar characteristics (such as age and sex), which can increase study efficiency. Wellcome Trust Case Control Consortium, for example, uses a case–control design in order to study multiple major diseases with the careful use of a common control group (72). The case–control design has been widely combined with meta-analysis approaches to pool summary-level data from different research groups, such as the Psychiatric Genomics Consortium (73) and ENIGMA (74). However, the selection and matching steps may be prone to certain biases and confounding effects, such as selection bias and recall bias. Due to potential differences between study samples and the general population, the findings and statistics learned from case–control designs may not be perfectly generalizable. As neuroimaging data were frequently collected as secondary traits or endophenotypes in these biomedical studies, the case–control nature needs to be taken into account when inferring these imaging traits in statistical analyses.

In contrast, cohort studies recruit participants without screening for the outcome of interest. Participants are selected based on their characteristics or their willingness to volunteer. The outcome of interest is typically monitored over time to assess its occurrence, and the relationship between outcome and exposures can be evaluated at baseline (e.g., cross-sectional analyses) or in a longitudinal framework. For example, the UKB is a large, population-based cohort study (3, 55), and many cross-sectional analyses have been conducted based on baseline data from UKB. However, UKB is well known for its healthy volunteer selection bias and may not be a true representation of the general population (75). To deal with selection bias, reweighting-based methods could be used from a causal inference perspective (58, 76). These methods typically assume that volunteer bias can be explained by observed variables, such as socioeconomic status. In addition, missing data are also a known source of confounding in cohort studies, especially when the outcome of interest is not independent of the missing mechanism. Failing to address these biases may lead to confounding effects, biased statistical results, and misleading findings.

Moreover, when meta- or mega-analyses integrate data from different studies and cohorts, the study designs of these sources may differ. Ignoring such differences may lead to unexpected results in DI. For example, it may not be straightforward to specify a correct statistical inference framework when pooling data from a case–control and a cohort study. It is obvious that naive analyses that do not take into account the study design will lead to biased findings. Therefore, it is important to understand sampling mechanisms and to apply them appropriately for the desired objectives when designing and merging population-based studies.

As compared to observational studies, there are fewer experimental studies in population-based biomedical research. One of the reasons is that it is typically difficult and expensive to conduct experiments on a large number of subjects. However, experiments play a key role in advancing our understanding in biomedical data science. For example, well-designed task- or event-based fMRI experiments can help us understand the brain functional changes due to human behavior and interventions. In addition, sequential decision-making is also important for designing better follow-up stages in large-scale population-based studies. In summary, the sampling mechanism needs to be taken into consideration when interpreting and generalizing findings from observational studies. It is evident that large-scale experimental designs for NDA are seriously lacking in major publicly available data resources, and this issue will require greater attention in future biomedical data science research.

#### 3.2.2. Statistical parametric mapping.

There is a large literature on various SPM methods, which are used for two major NDA tasks: image reconstruction from imaged volumes within an individual subject and group analysis of images obtained from different subjects/groups. In both tasks, images are assumed to be registered to the same space. Below we briefly review conventional SPMs and their extensions.

SPM is a statistical technique for detecting changes in brain structure and function recorded during neuroimaging experiments within individual subjects or across groups. SPM has been implemented in popular neuroimaging software platforms including SPM (http://www.fil.ion.ucl.ac.uk/spm) and FSL (FMRIB Software Library; http://www.fmrib.ox.ac.uk/fsl). The technique consists of three key modules: (*a*) smoothing neuroimaging data spatially or temporally, (*b*) fitting voxelwise general linear models (GLMs), and (*c*) correcting for multiple comparisons using random field theory (RFT), false discovery rate (FDR), or permutation methods. Despite the popularity of SPM, there is a great need to extend it in three important directions.

The first direction is to address several major drawbacks of the Gaussian smoothing method, which may dramatically increase the numbers of false positives and false negatives (77). Moreover, for twin studies, Li et al. (78) showed that smoothing raw images can dramatically decrease statistical power in detecting environmental and genetic effects, which is critically important for imaging genetic studies. To address these drawbacks, researchers have proposed multiscale adaptive models to extend the propagation-separation method to a large class of parametric and semiparametric models for group analysis (46, 77–79). These multiscale adaptive methods dramatically increase the signal-to-noise ratio while preserving spatial details.

The second direction is to move from GLMs to more advanced statistical models. This development is primarily motivated by complex study designs, sampling bias, missing data, complex data objects, and complex relationships, as discussed in themes T4–T8. Simply applying GLMs to all scenarios in T4–T8 can easily lead to false positive and false negative results. In the era of large-scale neuroimaging studies, it is important to integrate and extend many packages in professional statistical software, including R (http://www.r-project.org), RStudio (http://www.rstudio.com), SAS (http://www.sas.com), and Python statsmodels (http://www.statsmodels.org), which may not be directly applicable to NDA without modification, so that they can handle many parametric, semiparametric, and nonparametric statistical models and their associated inference tools.

There are two ways of applying and extending these models in statistical software. The first is to apply the models to neuroimaging data, generate maps for various statistical results (e.g., *p*-values, parameter estimates, and diagnosis measures) across spatial locations (e.g., voxels, vertices, or pixels), and then perform multiple comparisons (below we discuss in detail how to correct for multiple comparisons). Minimum effort is required for all necessary technical developments. The second way is to explicitly incorporate the spatiotemporal structure discussed in T2 into different models and then correct for multiple comparisons. Some notable developments include multiscale adaptive regression methods for longitudinal neuroimaging data (80), spatially varying coefficient models (77, 81–83), quantile models (84, 85), and functional principal component analysis (PCA) (86).

Four remarks on different statistical models for modeling neuroimaging data are in order. First, most models for SPM can be regarded as an approximation to Equation 1 in order to disentangle the signals of interest, such as age, gender, or diagnosis. Second, most models for SPM can be formulated as an image deconvolution problem according to Equation 2. Third, although quantile methods have not been widely used in NDA, they improve our understanding of the conditional distribution of imaging measures on the spatial domain that may have nonlinear relationships with various predictors in Equation 1. Fourth, most functional data analysis (FDA) methods in statistics were developed primarily for 1D curves (67, 87), and there are major statistical and computational challenges to extending these FDA methods to 2D and higher-dimensional neuroimaging data.

The third direction is to develop statistical methods, including RFT, resampling methods, and FDR, to correct for multiple comparisons in NDA. Most RFT and resampling methods control for the familywise error rate by accounting for the spatiotemporal structure of raw neuroimaging data, as discussed in theme T2, whereas most FDR methods directly operate on uncorrected *p*-values without addressing T2. However, several FDR methods have recently been developed to control for FDR in multiple testing of spatial signals (88, 89). Although FDR is applicable to a larger class of statistical models beyond GLMs, it depends on the computation of uncorrected *p*-values, which is nontrivial in many cases.

Since the beginning of fMRI, RFT has dominated the field of NDA primarily due to the many seminal contributions of Worsley, Adler, Nichols, Taylor, and their collaborators (90–92). RFT has been widely used for voxelwise and cluster size inference in order to test for the intensity of an activation and for the significance of its spatial extent. Voxelwise RFT uses the expected Euler characteristic heuristic of random fields to approximate the *p*-value of the maximum statistic, whereas cluster size RFT uses the distribution of the maximum cluster sizes in a zero-mean stationary random field. However, current RFT results cannot meet important prerequisites for many advanced statistical models in NDA, for two primary reasons. First, most RFT results are limited to GLMs and some minor extensions (91). For more advanced models, substantial effort is required for the development of new RFT results. Second, most RFT results require strong assumptions including stationarity and high-order smoothness, which are often invalid for fMRI. Eklund et al. (93) have made two important observations in connection with this point: (*a*) that some key assumptions of RFT are invalid for fMRI, and (*b*) that the existing RFT can lead to inflated false positive rates for cluster size inferences.

Resampling methods primarily include permutation and bootstrap-based methods, both of which approximate the null distribution of test statistics conditional on the observed data. Although permutation testing has received some attention in NDA, it has not gained much attention in statistics lately due to computational and methodological challenges. Specifically, permutation methods require complete exchangeability under the null hypothesis, which can be problematic even for the simplest two-group comparison problem. Bootstrap-based methods, particularly wild bootstrap, have gained substantial attention in statistics due to their flexibility, theoretical basis, and good empirical performance, even though additional effort may be required for further development of good wild bootstrap methods and their application to different models. Theoretically, resampling methods like wild bootstrap have been shown to be valid conditional on data (94, 95). Practically, wild bootstrap methods have been successfully applied to NDA, including a heteroscedastic linear model for surface analysis (96), regression analysis of asynchronous longitudinal functional and scalar data (97), functional mixed models for longitudinal neuroimaging data (80), and statistical models for imaging genetics (98, 99).

As an illustration, an interesting study (100) recently examined the variability of different SPM analytical pipelines in the analysis of a single neuroimaging dataset by 70 independent teams. Sizeable variations in the final statistical results of the hypothesis tests were caused by all three modules of SPM. A surprising observation was that the spatial smoothness of fMRI was the strongest factor explaining such variation. Another study further evaluated the effect of different fMRI preprocessing pipelines on analytical results (101). Both studies called for the additional development of resources and methods for reducing the variability in preprocessing and analysis pipelines and the effect of this variability on analytical results.

#### 3.2.3. Object-oriented data analysis.

Here we briefly review OOD and its extensions. OOD analysis is a comprehensive statistical framework including estimation methods and statistical theory for the analysis of populations of complex objects (36, 63–65, 67). Some specific examples of complex objects given in T7 include elements of mildly non-Euclidean spaces, such as Riemannian symmetric spaces, or elements of strongly non-Euclidean spaces, such as spaces of tree-structured objects. A primary application of OOD in NDA is group analysis of complex objects extracted from neuroimaging data.

There are three classes of analytical procedures for OOD: (*a*) feature analysis, (*b*) extrinsic analysis, and (*c*) intrinsic analysis. The key ideas of feature analysis are to use some feature extraction functions to project random objects to Euclidean-valued variables and then apply the second and third modules of SPM to those variables. A key advantage of feature analysis is its computational efficiency. Moreover, Euclidean-valued variables projected from random objects can be biologically meaningful if their corresponding extraction functions have strong biological interpretations. We consider two examples. The first example involves treating diffusion tensors, which are 3 × 3 SPD matrices, as random objects. It is common to calculate several invariant measures of a diffusion tensor, such as FA, and then use SPMs to analyze FA images. In neuroscience, FA is an indirect measure of fiber density, axonal diameter, and myelination in WM. The second example involves treating a functional brain network as random objects and using feature analysis to understand its topological organization. Specifically, one may calculate various graph metrics (e.g., nodal centrality, network efficiency, or degree) of functional brain networks and then perform the group analysis of these metrics (102, 103). For instance, network efficiency describes how a brain network efficiently exchanges information. However, it is often nontrivial to develop a good feature extraction function with a strong neuroscientific interpretation considering that the feature vector may contain only partial information about the original object.

The key ideas of extrinsic analysis are to (*a*) embed the curved space where the object resides onto some higher-dimensional Euclidean space, (*b*) perform statistical inferences on random objects in the embedded Euclidean space, and (*c*) project results back onto the curved space. A key advantage of extrinsic analysis is its computational efficiency. Existing extrinsic analysis methods have been developed for mean, median, local regression, and DR (104). For instance, diffusion tensors can be embedded in a six-dimensional Euclidean space, whereas the d-dimensional sphere $S^{d}$ can be embedded in the (d+1)-dimensional Euclidean space. The manifolds considered in directional statistics are spheres and projective spaces and the associated statistical tools are primarily extrinsic approaches. However, there are two drawbacks. First, it is nontrivial to propose a good equivariant embedding in most cases, which requires extensive thought and consideration. Specifically, in step *a*, equivariant embeddings are required to preserve a lot of geometry of the original curved space. Second, in many cases, it is unclear how to project results back onto the curved space.

The key ideas of intrinsic analysis are (*a*) to introduce a good metric ρ for the curved space ℳ where the object resides, denoted (ℳ,ρ), and (*b*) to perform statistical inference on random objects in (ℳ,ρ). Examples of metric spaces with additional structure include Riemannian manifolds, normed vector spaces, length spaces, and graphs. For instance, a Riemannian manifold (ℳ,g) is a real, smooth manifold ℳ equipped with a Riemannian metric tensor g defined for all tangent vectors at every point. One can define the geodesic distance between two points on a connected Riemannian manifold. We can further construct quotient metric spaces for (ℳ,ρ) based on an equivalence relation on ℳ, denoted ∼, by endowing the quotient set ℳ/∼ with a pseudometric $\rho_{P}$.

A fundamental issue in intrinsic analysis is how to appropriately introduce a good metric ρ for (ℳ,ρ) or a good metric tensor g for (ℳ,g). The choice of ρ (or g) has significant implications on downstream computation and statistical inference. For instance, Dryden et al. (105) discussed eight metrics of the space of SPD matrices for estimating the mean diffusion tensor. Recently, Srivastava & Klassen (36) introduced a general elastic metric, which includes the Fisher–Rao metric as a special case, for the shape analysis of curves, allowing us to separate phase and amplitude components. In general, the choice of ρ (or g) should focus on the signal of interest and data variability in random objects, while considering computational efficiency.

In the last decade, there has been significant progress in the development of intrinsic statistical models for manifold-valued data in finite-dimensional Riemannian manifolds. Fréchet mean, median, and variance provide a simple way of characterizing the center and variability of random objects in ℳ (64, 65, 106). Principal geodesic analysis (107) was further developed to reduce the dimensionality of random objects, while increasing interpretability and minimizing information loss. Cornea et al. (66) developed an intrinsic regression model based on Riemannian logarithm and exponential maps for random objects in a Riemannian symmetric space. Other notable contributions include intrinsic local polynomial regression (108), Riemannian FDA (109), Wasserstein regression (110), and a generic measure of dependence (111). Despite these new developments, computing intrinsic estimators is notoriously difficult, which requires further attention.

Statistical shape modeling and analysis have emerged as important tools for understanding brain structure and function extracted from neuroimaging data. Four key components of shape analysis are (*a*) shape representation, (*b*) distance between shapes, (*c*) shape registration, and (*d*) group analysis of shapes. Shape analysis methods depend on shape representations including landmarks, implicit representations, parametric representations, medial models, and deformation-based descriptors (34, 36, 40, 63, 64, 112, 113). Most earlier representations focus on either points on the object boundary or parametric descriptors of the object boundary, whereas deformation-based representations use shape information in the entire image. Most shape spaces are quotient metric spaces based on an equivalence relation, including translation, rotation, and scaling. Some notable shape analysis methods include the large deformation diffeomorphic metric mapping technique (34), elastic statistical shape analysis (36, 114), and Wasserstein shape analysis (115).

#### 3.2.4. Imputation methods.

Developing good imputation methods for neuroimaging data requires a solid understanding of the mechanisms of missing data in NDA and their causes. Table 1 summarizes some common reasons for missing data and their corresponding missing mechanisms in NDA. Reasons for missing data in NDA include missing image modalities due to different acquisition protocols, different study designs, data transfer and storage loss, faulty scanning due to image corruption and susceptibility artifacts, and participant attrition due to allergies to materials, personal beliefs, and financial costs, among others. There are three missing mechanism categories: MCAR, MAR, and MNAR (61, 62). Distinguishing between MAR and MNAR depends on whether the missingness is predictable based on either observed covariates or a missing variable itself. For example, if dropout rates differ according to observed covariates (e.g., age, sex, or race), then the missing mechanism is traceable and therefore MAR. In contrast, if dropout depends on missing data itself, then it is MNAR and ignoring such missingness may introduce substantial bias. MCAR, as a special case of MAR, assumes that the distribution of the missing data is indistinguishable from the nonmissing data. Such an assumption is strong and usually difficult to meet in practice. In general, when values are missing systematically, conducting downstream data analysis without correcting for missing data may lead to erroneous conclusions.

There are at least two main strategies for handling missing data: omission and imputation (61, 62, 116). Common omission approaches include listwise/pairwise omission and feature dropping. Although omission is simple and easily used, it can lead to serious estimation bias, a large loss in efficiency, and a dramatic reduction in statistical power. There are two types of imputation methods: single imputation and multiple imputation. Single-imputation methods generate one imputation value for each missing observation, which leads to a single complete dataset that treats the imputed values as the true values in downstream data analysis. Therefore, downstream analyses based on the single-imputed complete dataset do not account for the imputation uncertainty. The two main strategies of single imputation are imputation by statistical values (e.g., mean, median, or maximum) and imputation by predicted values generated from a statistical model. Multiple-imputation methods generate many imputed values for each missing observation, which leads to many complete datasets to be analyzed in downstream data analyses. Multiple-imputation methods allow one to explicitly account for imputation uncertainty.

Some additional statistical challenges arise from handling missing neuroimaging data due to themes T1–T4, even though both omission and imputation methods are useful for NDA. Specifically, as discussed in Section 3.1 and Figure 4, image data are largely blockwise missing when there is a large number of features across different domains (e.g., genetics/genomics) in various biomedical studies. In this case, missing data requires building image imputation models to impute missing data in high-dimensional images conditional on all other observed features, which may include data from other imaging modalities, genetic/genomics data, and demographic data. One promising research topic is to develop deep generative models, which have been used to achieve impressive results in image generation and image-to-image translation for image imputation models. In particular, image-to-image translation is designed to learn the mapping between an input image and an output image while preserving the content representation (117). This task can be further classified into paired and unpaired imputation according to whether both input and output images are available on the same subjects in the training data. For instance, conditional generative adversarial network (CGAN) methods, such as the pix2pix (118) method, perform pixel-to-pixel image synthesis using paired image data, whereas CycleGAN (119) was developed to model image translation based on unpaired data. Although many image-to-image translation models for specific neuroimaging pairs have been developed, these models require substantial validation efforts and the use of synthetic and real datasets for downstream tasks such as prediction. Furthermore, it is interesting to incorporate additional information (e.g., genetics, diagnosis status, and sex data) to impute missing image data, while imposing their dynamic causal relationships shown in Figure 1. However, there has been little work in this direction on the development of CGAN-based imputation models for neuroimaging data. In addition, since the missing mechanism of image data may be MNAR, as detailed in Table 1, it is important to develop CGAN imputation models under MNAR.

#### 3.2.5. Data integration.

We have witnessed an exponential increase in the collection and availability of multiview data from different studies and clinics, including electronic health records, imaging data, genetic data, sensor data, and text. DI is the process of integrating multiview data from different sources into a unified view of information for better data management and downstream analyses. A good DI system consists of (*a*) a feature engineering pipeline for generating more complete high-quality data and their associated features, (*b*) SL methods for DI associated with different NDA tasks, and (*c*) a feedback loop to improve data collection and feature extraction for major NDA tasks. The feature engineering pipeline consists of data ingestion, data processing, data annotation, data transformation, and data storage. Missing data imputation applies in all of these tasks, whose related methods were discussed in Section 3.2.4. However, although much progress has been made in the last decade, it remains challenging to develop a good DI system for NDA due to the fact that the data are complex, heterogeneous, temporally dependent, irregular, poorly annotated, and generally unstructured, as discussed in themes T1–T8.

Here we review SL methods for DI within and across individual studies that are associated with four major NDA tasks, including (*a*) multimodal neuroimaging fusion, (*b*) the genetic architecture of neuroimaging measures, (*c*) gene–environment interaction on neuroimaging measures, and (*d*) the CGIC pathways. We refer the reader to Section 3.2.7 for a discussion of most SL methods for tasks *b* and *c*, and to Section 3.2.8 for a discussion of SL methods for task *d*. Popular building blocks in SL methods for DI include feature concatenation, Bayesian methods, tree-based ensemble methods, multiple kernel learning, matrix/tensor factorization, and DL (120, 121). For instance, Bayesian methods can easily incorporate prior information from different views, whereas tree-based methods can use ensemble methods to integrate trees learned from each view.

As an illustration, we consider matrix factorizations and DL for DI in a single study. First, we consider a generic model for multiview integration in a single study. Suppose that we observe a $p_{k}\times n$ row-mean-centered data matrix, denoted $I_{k}$, for the k-th view of K views on n subjects, where $p_{k}$ is the number of variables. A generic model for matrix/tensor factorizations is given by

3. $$I_{k}=C_{k}+D_{k}+E_{k}\;\;for\;\;k=1,\ldots ,K,$$

where $C_{k}$ is a low-rank common-source matrix representing latent factors common across all views, $D_{k}$ is a low-rank distinctive-source matrix representing distinctive latent factors of the corresponding view, and $E_{k}$ is the noise matrix. Some state-of-the-art matrix factorization methods based on Equation 3 include common orthogonal basis extraction (122), the JIVE (joint and individual variation explained) method (123), and decomposition-based generalized canonical correlation analysis (124). These methods differ in how they reconstruct the common- and distinctive-source matrices.

Second, we consider the hierarchical architecture of DL for multiview integration as another powerful method. Its hierarchical structure consists of (*a*) the construction of subnetworks $s_{k}={𝒩}_{k}\left(I_{k}\right)$ (e.g., variational autoencoders and generative adversarial networks for neuroimaging data) for k=1,…,K, and (*b*) the integration of all individual subnetworks into a model $Y=f\left(s_{1},\ldots ,s_{K};\theta \right)+\epsilon$, where f(⋅) is a link function, θ is a vector of parameters, and ϵ is an error term. We can use an objective function similar to Equation 2 to tune θ and $\left\{{𝒩}_{k}\right\}$. Miotto et al. (125) discussed different architectures of subnetworks for individual views. These subnetworks can be first adopted from some pretrained models from other fields, such as computer vision, and then tuned in the whole model at the integration stage.

We consider two major methods for DI across multiple studies or centers: the merged learner and the ensemble learner methods. The merged learner proceeds with merging and processing data from all studies and then training a single learner based on the merged data. It is common to use fixed- or random-effect models to train the learner (126). The ensemble learner proceeds with training a learner based on the data obtained from each study and then uses a weighted average of all learners. It includes ensemble machine learning (127), meta-analysis (128), fusion learning (129), and federation learning (130). ENIGMA has been using the ensemble learner in most of its imaging genetic studies, but it has started to use the merged learner (or mega-analysis) (126). Since data pooling can dramatically increase sample size and ensure consistent data processing and quality control, the merged learner method will be increasingly used in international neuroimaging efforts.

There are two major issues in mega-analysis: heterogeneity within individual subjects and across centers/studies (theme T4) and sampling bias (theme T5). First, there is a great interest in developing data harmonization methods to explicitly correct additive site and scanner effects, covariance batch effects, hidden factors, and some structural priors in neuroimaging data (30, 31, 131). These methods partially remove the effects of confounding variables that are not of interest, but they require extensive validation using walking phantoms and synthetic and annotated datasets. Second, although it is tempting to pool multiview data from studies with different study designs, simple statistical methods based on fixed- and random-effect models (132, 133) cannot appropriately handle such issues. There are several key problems. First, in many imaging-related studies (e.g., ADNI and UKB), neuroimaging data are only the secondary phenotypic variables, so it can be very problematic to not adjust sampling bias even in a single study (134, 135). Second, many neuroimaging-related studies have different study designs and may have minimal overlap in key confounding variables of interest (e.g., age). For instance, besides the age differences across HCP and ADNI, there are many twins in HCP, whereas ADNI has many longitudinal observations. This raises many serious issues concerning the target population for the merged sample, the type of scientific questions to be answered, and the choices of statistical models (e.g., prospective and retrospective likelihood). In conclusion, one cannot simply perform the merged learner method for many NDA tasks without appropriately addressing sampling bias (theme T5).

#### 3.2.6. Dimension reduction methods.

The goal of DR is to transform data from a high-dimensional space to a relatively low-dimensional space, while retaining important information in the original data. There is a large literature on the development of various statistical methods for DR due to theme T3. We can group DR methods into feature selection and feature extraction methods. Feature selection aims to find a subset of the original features for a specific task, whereas feature extraction aims to construct new features from the original features. Originally, the above-mentioned DR methods were developed to solve the small-*n*-large-*p* problem, where the number of subjects is much smaller than the number of imaging variables. However, with the availability of many large-scale neuroimaging studies, we have to deal with the large-*n*-large-*p* problem, in which both the number of subjects and the number of variables are both extremely large. This large-*n*-large-*p* problem requires further developments in DR methods.

The feature selection methods can be further grouped into the filter strategy, the wrapper strategy, and the embedded strategy based on how the selection algorithm and the model building are combined (136). Filter methods use a selection measure, such as correlation and distance correlation, to select a feature subset. Wrapper methods, such as stepwise regression, use a search algorithm based on a predictive model to score feature subsets. Embedded methods, such as decision tree and LASSO (least absolute shrinkage and selection operator), select features as part of the model construction process. In practice, feature selection is essential to eliminate a large number of noisy variables before running downstream data analysis.

The feature extraction methods can be categorized into knowledge-based and data-driven approaches. In NDA, knowledge-based feature extraction uses specific human brain atlases to perform feature extraction within individual regions and across region pairs. The use of several tens to several hundreds of homogeneous regions of interest (ROIs) in brain atlases dramatically reduces the complexity of multiple neuroimaging datasets. This improves neuroanatomical precision for studying the structural and functional organization of the human brain. The data-driven feature extraction methods can be grouped into unsupervised, supervised, and semisupervised approaches for both traditional approaches and modern DL (137, 138). Some notable examples of unsupervised feature extraction methods include PCA, kernel PCA, functional PCA, single value decomposition, tensor decomposition, multidimensional scaling, and independent component analysis. Readers are referred to Reference 137 and references therein for a systematic review and empirical comparisons of various unsupervised DR approaches. Some notable examples of supervised DR methods include linear discriminant analysis, partial least squares regression, and canonical correlation analysis. Feature extraction and feature selection methods have been integrated together to solve the small-*n*-large-*p* problem, while accounting for complex spatiotemporal structures (theme T2) (139, 140). However, while most existing feature extraction methods are infeasible for the large-*n*-large-*p* problem due to limited computing speed and computer memory, several hierarchical feature extraction methods have been developed to address related challenges (141, 142).

There are three classes of unsupervised DL approaches (or the SSL approach) to extracting image embeddings: generative, contrastive, and adversarial (138). These SSL approaches train the encoder–decoder networks by encoding input images into a low-dimensional representation, contrasting semantically similar and dissimilar pairs of embeddings, and generating fake samples that a discriminator can hardly distinguish from real samples. Recently, semisupervised SSL approaches have been developed by incorporating downstream tasks, such as classification or prediction, into the original pretext tasks (construction and contrasting) (143). Compared with traditional DR approaches, DL-based DR approaches usually extract more informative representations by taking advantage of increased computing power and more flexible frameworks.

#### 3.2.7. Imaging genetics.

The genetic architectures of human brain structures and functions are of great interest. Using imaging traits as phenotypes, previous family- or population-based studies have quantified the extent to which genetics can affect the structure and function of the human brain (or heritability) (144, 145). Several consortia, such as ENIGMA (74), CHARGE (Cohorts for Heart and Aging Research in Genomic Epidemiology) (146), and IMAGEN (147), were established to discover the genetic loci associated with human brain structures. In recent years, large-scale MRI datasets, such as UKB and Adolescent Brain Cognitive Development (ABCD), have provided further insights into the genetic determinants of the human brain. For example, Elliott et al. (148) and Smith et al. (149) screened more than 3,000 brain functional and structural imaging phenotypes from the UKB study. The genetic architecture of commonly used imaging traits, such as the regional gray matter volumes from sMRI (150), WM microstructure from DWI (151), and functional connectivity from fMRI (152), have been discovered. From these studies, hundreds of brain-related genetic loci have been identified, and substantial genetic overlaps with major brain disorders were observed, such as AD and schizophrenia. Several open resource knowledge portals have been developed in imaging genetics, including the Oxford BIG40 (https://open.win.ox.ac.uk/ukbiobank/big40/) and BIG-KP (Brain Imaging Genetics Knowledge Portal; https://bigkp.org/). While they extract imaging features using distinct pipelines, these knowledge portals provide similar findings regarding the genetic control of the human brain. Figure 3b presents the heritability of various imaging phenotypes based on UKB.

A typical imaging genome-wide association study (GWAS) contains the following steps. First, we develop or apply imaging data analysis pipelines to extract imaging features from raw neuroimaging data. For example, in the WM GWAS (151), we applied the ENIGMA-DTI (diffusion tensor imaging) pipeline to extract WM microstructure measures from over 40,000 subjects (153). Although voxelwise or vertexwise feature maps are available, aggregate imaging traits at the brain region level (such as ROIs and WM tracts) are typically used in subsequent genetic discoveries. In addition to improving the signal-to-noise ratio, these region-level traits may reduce the burden of multiple testing, while increasing the statistical power in genetic analysis. Second, variant-level and gene-level association analyses can be performed to detect significant genetic variants or genes in a large-scale discovery cohort. An independent holdout cohort, which is typically smaller than the discovery one, will be used to examine if the significant associations between trait and gene/gene variant can be replicated. Further replications and generalizability can be explored using racially diverse cohorts. Additionally, polygenic risk scores can also provide evidence of validation by evaluating the proportion of variance of imaging traits that can be predicted by genetic variants.

A few tools have been developed to estimate the heritability using individual-level [e.g., GCTA-GREML (genomic-relatedness-based restricted maximum-likelihood–genome-wide complex trait analysis) (154)] or summary-level [e.g., univariate LDSC (linkage disequilibrium score regression) (155)] data. Furthermore, partitioned LDSC can be used to estimate the enrichment of heritability related to specific brain tissue or cell types, such as glia and neurons. FUMA (functional mapping and annotation) (156) is a useful platform for functional gene mappings based on summary-level data. The coloc, bivariate LDSC, and Mendelian randomization methods (157) can quantify the genetic relationships between imaging traits and other complex traits or diseases from different perspectives. Readers are referred to Reference 158 for a recent review of GWAS methods.

Despite significant recent advancements in imaging genetics, it remains challenging to map the causal biological pathways linking genetics and brain abnormalities to neuropsychiatric disorders (13, 159) (see Figure 1b for a hypothetical causal pathway). Neuroimaging can identify important endophenotypes in the causal pathway by which genetic variation impacts risk for brain diseases. The identified genetic loci in large-scale imaging genetic cohorts need to be integrated with multiple layers of biomedical data, such as RNA, proteins, brain cells, and brain tissues (71). It is necessary to make greater efforts to collect and integrate multiple types of biomedical data and develop better statistical models for causal analysis (160). Clinical applications can also benefit from recent imaging genetic discoveries. For example, the combination of genetic polygenic risk scores and MRI data could provide better predictions of the risk of brain diseases (161).

#### 3.2.8. Causality research.

Causality research has received a lot of attention in neuroscience research (71, 159–169). Some important scientific questions in neuroscience include how do experimental stimuli affect brain function, how are different brain regions causally linked in a specific task, how are brain structure and function causally linked, how does brain structure mediate the relationship between genetics and clinical variables, how does brain structure mediate the relationship between therapies/drugs and clinical variables for brain-related diseases, and what are the causal relationships between genetics, brain, health factors, and brain disorders? Addressing these questions raises serious challenges in experimental design, data collection, DI, unobserved confounders, SL methods for causal research, and causality validation. For instance, although randomized controlled trials (RCTs) have been widely regarded as the gold standard for causal discovery, it may be inappropriate to run RCTs in many neuroscience scenarios due to ethical or practical reasons. Therefore, one may have to draw causal conclusions from existing observational data under a series of strict assumptions.

Causality research can be roughly divided into causal discovery for determining causal relationships among a set of variables and causal inference for estimating causal effects deriving from a change of a certain variable over an outcome of interest in a large system (170–174). Causality research proceeds with the development of the causal models (e.g., the CGIC pathway in Figure 1) for a set of variables with possibly unobserved confounders. The three main causal models are the Bayesian network (BN) model based on a directed acyclic graph (DAG), the structural causal model (SCM) given a DAG, and the Rubin causal model (RCM). These causal models complement each other and have their own pros and cons. Under some conditions, SCM is a causal BN model, while RCM is logically equivalent to SCM (171). SCM and BN are more popular in computer science and epidemiology since they offer a graphical representation with reasonable interpretability and explainability. In contrast, RCM is very popular in statistics, economics, and social sciences since it is well connected with experimental design and causal effect estimation.

The causal discovery methods for causal BN models can be categorized into discrete space algorithms and continuous space algorithms (173). Traditional discrete space algorithms, including constraint-based and score-based methods, search for the optimal graph from a discrete space of candidate graphs by using either statistical tests or scores (e.g., Bayesian information criteria) to estimate the causal structure of a DAG. In contrast, continuous space algorithms find an optimal graph from the continuous space of weighted DAGs based on machine learning algorithms. Computationally, the complexity of traditional discrete space algorithms grows with the number of nodes in DAG, whereas continuous space algorithms are more scalable. Moreover, causal discovery methods are designed for three types of data under analysis, including cross-sectional, time-series, and longitudinal data. Cross-sectional and time-series data are distinguished in that, in time-series data, there is a time component so that events in the present cannot cause events in the past. The Granger causality method is one of the well-known methods for performing causal discovery for time-series data.

As an illustration, we consider different causal discovery methods for using functional neuroimaging data (e.g., fMRI) to infer effective connectivity, which is a causal model of the interactions between ROIs. Different discrete space algorithms and their extensions have been used for effective connectivity (175). Other statistical methods for effective connectivity include Granger causality, dynamic causal models, structural equation models, state-space models, RCMs, directed graphical models, and dynamic BN models (162–165). However, most existing network methods suffer from large estimation errors for connection directionality (169).

We estimate the causal effect of a specific treatment (X) over a certain outcome of interest (Y) in two steps: (*a*) the study of identification questions for X→Y and (*b*) the estimation and inference methods for the causal effect X→Y. Specific identification strategies for step *a* include experimental design, adjustment/unconfoundedness, instrumental variables, difference-in-differences, regression discontinuity designs, synthetic control methods, and causal mediation analysis. For instance, it is common to use the front-door and back-door criteria to identify valid adjustment sets (171, 173, 174). Causal inference algorithms only work when all common causes of X and Y have been included in observational data (called causal sufficiency), so controlling unobserved confounding requires a series of strong assumptions (176, 177). In step *b*, SCM explicitly specifies all mediators, whereas RCM does not handle unspecified mediators in the outcome-generating model.

As an illustration, we consider the integration of multiview data from ADNI to infer a hypothetical causal model for biomarker dynamics in AD pathogenesis, as presented by Jack et al. (178). It starts from AD risk genes for the abnormal deposition of β-amyloid fibrils, which leads to increased levels of cerebrospinal fluid tau protein, hippocampal atrophy, declined cognitive symptoms and impairment, and AD. Existing SL methods focus on associations between different views, but there is a growing interest in delineating the temporal causal relations in Jack et al.’s causal model—say the causal effect of hippocampal atrophy (X) on behavioral deficits (Y) (160). Our CGIC pathway (Figure 1b) is an approximation of Jack’s causal model. We need to check the causal sufficiency of X and Y, which is most likely invalid in practice. Although there are several popular identification strategies, including instrumental variables and the front-door criterion, for handling the issue of unobserved confounding, each of them has to make some strict assumptions. For instance, Mendelian randomization is an instrumental variable method, which selects a set of genetic variants (G) as instruments to estimate the causal effect of X→Y (157). It requires three key assumptions including relevance, independence, and no horizontal pleiotropy. It can be implemented using individual-level data in a single sample or summary data from two samples. Several popular instrumental variable estimation methods include the ratio method, two-stage methods, likelihood-based methods, and semiparametric methods (176, 177). Furthermore, it is of great interest to build SCMs to link all variables in ADNI together and infer their time-varying causal relationships by extending causal mediation methods (179). This is motivated by delineating how most brain-related disorders progress, adjusting for temporal confounding by various health factors (71).

#### 3.2.9. Predictive models.

There is a large literature on the development of SL methods for building predictive models in neuroscience and clinical translational research (7, 180–182). The goal of a predictive model is to use a set of current and historical features x to predict future events in Y. This is motivated by the identification of biomarkers (e.g., neuroimaging) that could aid in detection, diagnosis, prognosis, prediction, and monitoring of disease status, among many other objectives. As shown in Figure 1, the feature vector x in NDA may include neuroimaging, genetic, environmental, and demographic variables, while Y is a low-dimensional vector consisting of data on cognitive scores, diagnosis, and survival times, among others. Despite the fact that much progress has been made recently in academic settings, most predictive models in NDA have not been adopted in clinical practice.

A good predictive system in NDA for clinical translation includes (*a*) a feature engineering pipeline to generate cost-effective and reliable biomarkers (e.g., blood) and perform high-quality data annotation; (*b*) SL methods for training predictive models with high predictive capacity, robustness, and clarity for the main NDA tasks; and (*c*) a feedback loop to improve tasks *a* and *b*. Developing a good predictive system requires appropriately handling themes T1–T8, among which T4 needs closer attention. Equation 1 emphasizes that neuroimaging data contain external heterogeneity caused by exogenous factors (e.g., the device, acquisition parameters), as well as internal heterogeneity associated with downstream tasks for Y (183). Specifically, “internal heterogeneity” refers to how diseased regions may significantly vary across subjects or time in terms of their numbers, sizes, degrees, and locations. A good predictive system has to appropriately handle both external heterogeneity and internal heterogeneity in neuroimaging data through further developments in tasks *a* and *b*, among which *a* is the biggest bottleneck.

Existing SL methods for predictive models in NDA have various pros and cons. First, most existing supervised learning and variable selection methods (182) are suboptimal for predictive models in NDA due to the nonsparse effect of image biomarkers on Y and T4 in neuroimaging data. Second, DL methods (184) have achieved very promising results when handling pattern recognition problems, including the issue of internal heterogeneity in neuroimaging data discussed above. Training good predictive models requires large-scale representative datasets with high-quality data annotation. Third, it is interesting to develop SL methods for causal predictive models in NDA, which use causal thinking to improve prediction modeling (170, 171). Specifically, we might test and validate the dynamic causal relationships in Figure 1 based on observational data and then incorporate such causal findings to estimate risk under hypothetical interventions.

### 3.3. Challenges

We have reviewed the nine important PSA techniques, most of which represent emerging fields and pose several statistical challenges. First, large-scale neuroimaging-related datasets are too complex for most research teams in academia and industry and require a close multidisciplinary collaboration among experts with strong skills in statistics, biostatistics, epidemiology, genetics/genomics, engineering, applied mathematics, machine learning, neuroscience, brain disorders, imaging physics, and imaging analysis. Second, it is very difficult to appropriately process data across different domains with high quality, while controlling for potential biases introduced during the preprocessing stage. This requires the scientific community to work closely together to test all major preprocessing tools for reproducibility, generalizability, and reliability using well-designed synthetic and real datasets. Third, it remains uncertain how to appropriately integrate data across different domains obtained from different studies and cohorts with potentially different study designs without introducing biases. Although one might attempt to integrate as many variables and studies as possible in a project, this would likely lead to serious biases in downstream data analyses and conclusions. Fourth, it remains unclear how to appropriately and efficiently analyze neuroimaging-related datasets with multiple V’s (e.g., volume, velocity, variety, and veracity) while ensuring algorithmic fairness. Many existing statistical and machine learning models were developed before the era of big data, so they might make strong assumptions that are inappropriate for neuroimaging-related datasets, as discussed in Sections 2 and 3. We expect that many novel SL methods for NDA will be developed in the next decade.

## Supplementary Material

Appendix

Supplementary Tables

## Figures and Tables

**Table 1:** Scenarios with different missing data mechanisms in cognition- or behavior-related studies

Missing mechanism	Causes	Details
MCAR	Faulty scanning	Removal of images with corruption or susceptibility artifacts
	Faulty scanning	Random failure of experimental instrument
	Data loss	Data transfer/storage loss
	Data loss	Missing entries
	Attrition/nonresponse	Participants unable to participate due to migration/move (irrelevant to the study)
	Study design	Study ended early
	Study design	Modalities were not included in the imaging protocol
MAR	Study design	Exclusion criteria, such as age, sex, race, and socioeconomic status
	Attrition/nonresponse	Participant dropout due to side effects, such as allergic reactions
	Attrition/nonresponse	Participant dropout rates vary among different age or sex groups
MNAR	Study design	Participants quit the study due to physical or psychological health conditions
	Attrition/nonresponse	Participant dropout due to concerns of financial cost
	Attrition/nonresponse	Participant dropout due to concerns of limited available time to visit
	Attrition/nonresponse	Participant dropout due to concerns of scanning safety
	Attrition/nonresponse	Participant dropout due to concerns of unauthorized disclosure of personal data
	Attrition/nonresponse	Participants quit the study following another person’s behavior
	Attrition/nonresponse	Participants deliberately unwilling to respond

Abbreviations: MAR, missing at random; MCAR, missing completely at random; MNAR, missing not at random.
