# Supplementary material for: Statistical Learning Methods for Neuroimaging Data Analysis with Applications
Source: Annu Rev Biomed Data Sci. Author manuscript; Available in PMC 2025 Apr 2. (PMC11962820; doi:10.1146/annurev-biodatasci-020722-100353)
Supplement: Appendix [file NIHMS2065436-supplement-Appendix.pdf]

Supplemental Material: Annu. Rev. Biomed. Data Sci. 2023. 6:73-104
https://doi.org/10.1146/annurev-biodatasci-020722-100353

Supplementary Appendix for "Statistical Learning Methods for Neuroimaging Data Analysis with Applications"

Hongtu Zhu[1], Tengfei Li[2], and Bingxin Zhao[3]

[1]Departments of Biostatistics, Statistics, Genetics, and Computer Science and Biomedical Research Imaging Center, University of North Carolina, Chapel Hill
[2]Departments of Radiology and Biomedical Research Imaging Center, University of North Carolina, Chapel Hill
[3]Department of Statistics and Data Science, University of Pennsylvania

Downloaded from www.annualreviews.org. Guest (guest) IP: 152.19.89.75 On: Wed, 12 Mar 2025 14:59:29

Downloaded from www.annualreviews.org. Guest (guest) IP: 152.19.89.75 On: Wed, 12 Mar 2025 14:59:29

S1 A Review of Neuroimaging Techniques and Uses

For each image modality, we describe its tracer, data dimension, features, main uses, and several key softwares.

- *Structural magnetic resonance imaging (sMRI)* measures the fluid characteristics of different tissues (gray and white matter), creating high-resolution (0.5mm-1mm) images with a strong gray/white matter contrast and many anatomical details. It allows us to qualitatively and quantitatively measure the development and change of cortical and subcortical structures in terms of both size and shape in the brain. Some sMRI derived measurements include cortical thickness, cortical folding, sulcal depth, voxel-based morphometry, and regional volumes and shape. sMRI has been widely used for diagnosis, staging, and follow-up of disease in clinics and brain development in research.

- *Diffusion weighted MRI (DWI)* measures the Brownian motion of water molecules within voxels, creating images with a relatively low spatial resolution of 1.25-3 mm and multiple $b-$values and tens to a few hundreds of diffusion directions that can reveal microscopic details about tissue architecture and map white matter trajectories in the brain. It allows us to qualitatively and quantitatively measure white matter (WM) trajectories and water diffusivity along these trajectories *in vivo*. Some DWI derived measurements include invariant measures (e.g., fractional anisotropy) along WM trajectories (or in WM regions) and their related weighted and binary network metrics (e.g., the counts of streamlines connecting all WM region pairs). DWI has been used for delineating tumors, suspected acute ischemic brain injury, intracranial infections, masses, trauma, and edema in clinics and mapping structural connectome in research.

Downloaded from www.annualreviews.org. Guest (guest) IP: 152.19.89.75 On: Wed, 12 Mar 2025 14:59:29

- *Functional MRI (fMRI)* primarily measures the blood-oxygen-level-dependent (BOLD) responses in blood flow associated with brain function, creating images with a typical spatial resolution of 3-4 mm, a typical temporal resolution of 1-3 s, and hundreds of time points that can map metabolic function and neuronal activity. It allows us to indirectly and non-invasively measure brain functions under specific tasks, resting state, and naturalistic paradigms. Thus, fMRI consists of task based fMRI (tfMRI) and resting state fMRI (rsfMRI). Some fMRI derived measurements include voxel-wise activation patterns (e.g., beta images), region-based activation and interaction patterns, and weighted and binary network metrics (e.g., the correlation matrix for all region-of-interest pairs). fMRI has been used for brain activity mapping under different tasks, brain abnormalities detection, and pre-operative mapping of brain functions.

- *Positron emission tomography (PET)* measures emissions from radioactive tracers (e.g., 18F-FDG), creating images with a spatial resolution of 4-5 mm, and a poor temporal resolution (tens of seconds to several minutes) that can reveal tissue's metabolism (e.g., flow, oxygen, and glucose metabolism). It allows us to qualitatively and quantitatively measure the physiology and anatomy of brain as well as its biochemical properties. Some PET-derived measurements include voxel-wise activation patterns (e.g., standard uptake ratio (SUR) images) and region-based activation and interaction patterns. PET has been used for mapping brain functions and detecting abnormalities in brain neurophysiology and neurochemistry associated with Alzheimer's Disease, anxiety, and stroke.

- *Computerized tomography (CT)* measures X-ray attenuations by different tissues inside the body, creating images with a high spatial resolution of tens of nanometers that can non-destructively reveal internal details (e.g., soft tissues or bones) of organs. It allows us to qualitatively and quantitatively measure internal organs, bones, soft tissue, and blood vessels. However, CT as a radiation diagnostic technique can cause

Downloaded from www.annualreviews.org. Guest (guest) IP: 152.19.89.75 On: Wed, 12 Mar 2025 14:59:29

adverse effects, including harmful tissue reactions and cancer. Some CT derived measurements include local and regional volumetric and thickness measures and various radiomic features, including first-order features, second-order textural features, shape descriptors, and wavelet-based features. CT has been used for diagnosing a range of conditions, such as abnormal blood vessels, brain atrophy, head injuries, hemorrhage, swelling, stroke, and tumors.

- *Electroencephalography (EEG)* measures the electrical field produced by neuron electrical activity, creating an electrogram of the electrical activity on the scalp with a high temporal resolution of millisecond or less. It allows us to indirectly and non-invasively measure synchronous dendritic activity of cortical pyramidal neurons. However, EEG has a poor spatial resolution of 5-40 cm^3, which depends on the number of electrodes ranging from tens to a few hundreds. Some EEG derived measures include event-related potentials (e.g., stimulus onset) linked to an event, connectivity measures, network measures, and the type of neural oscillations in the spectral content of EEG, including delta (1–4 Hz), theta (4–8 Hz), alpha activity (8–12 Hz), beta (13–30 Hz), low gamma (30–70 Hz), and high gamma (70–150 Hz). EEG has been used for diagnosing and treating brain tumors, brain damage, brain dysfunction, sleep disorders, anxiety, epilepsy, inflammation, and stroke.

- *Magnetoencephalography (MEG)* measures the magnetic field produced by neuron electrical activity, creating an electrogram of the electrical activity with a high temporal resolution of millisecond or less and a moderate spatial resolution of a few millimeters. Compared with scalp EEG, MEG uses very sensitive magnetometers to indirectly and non-invasively measuring the tangential components of post-synaptic intracellular currents in the dendrites of neurons. MEG and EEG share similar derived measures. MEG has been used for identifying the functional areas of the brain, including centers of sensory, motor, language, and memory activities, and for mapping the precise

location of the source of epileptic seizures.

- *Functional near-infrared spectroscopy (fNIRS)* uses infrared light (650–900 nm) to measure changes in cortical BOLD response associated with brain function, creating an electrogram of BOLD signals with a high temporal resolution of milliseconds and a spatial resolution of millimeters below cortical surface. fNIRS shares similar derived measures with EEG and fMRI. fNIRS has been used for studying normal and pathological brain physiology and investigating behavioral and cognitive development in infants and children.

Downloaded from www.annualreviews.org. Guest (guest) IP: 152.19.89.75 On: Wed, 12 Mar 2025 14:59:29

S2 Large-scale Neuroimaging-related studies

S2.1 ADNI

The overall goal of ADNI is to validate potentially useful biomarkers for AD clinical treatment trials (1). ADNI is a multisite, prospective clinical study and actively supports the investigation and development of treatments that may slow or stop the progression of Alzheimer's disease (AD) *. Researchers across 63 sites in the US and Canada have been tracking the progression of AD through clinical, imaging, genetic and biospecimen biomarkers, starting from normal aging, early mild cognitive impairment (EMCI), late mild cognitive impairment (LMCI) to dementia or AD. The recruitment for ADNI was designed to mimic a clinical trial population, where the participants were generally well educated, mostly white, and with a high proportion of *APOE4* carriers among the MCI and AD groups, which is consistent with subjects in MCI and AD clinical trials. The ADNI participants do not represent "typical" subjects across the old population since the proportion of AD cases (23%) is much higher than the prevalence of AD in the US. Up till 2022, the ADNI study has collected 2,723 participants aged above 40 in four phases including ADNI1, ADNI2, ADNIGO, and ADNI3.

*ADNI, `https://adni.loni.usc.edu/study-design/`

Downloaded from www.annualreviews.org. Guest (guest) IP: 152.19.89.75 On: Wed, 12 Mar 2025 14:59:29

Genomic biosample and genotyping were collected for all subjects, enabling polygenic risk scores and gene pathway- and network-based metrics for prediction of disease progression. Cerebrospinal fluid (CSF) biomarkers, including the $A\beta$, t-tau and p-tau, were provided. For imaging data, ADNI1 focused primarily on sMRI and PET to study brain changes in brain morphology and metabolism with AD. ADNIGO and ADNI2 added a high-resolution coronal T2, perfusion MRI, DWI, and resting-state fMRI (rsfMRI). ADNI3 includes seven sequences (sMRI, FLAIR, T2*GRE, DWI, rsfMRI, ASL perfusion MRI, and a high resolution coronal T2 fast spin echo) in all subjects. ADNI3 aims to study how tau PET, CSF biomarker, and functional imaging affect treatment, promote the development of new immunoassay platforms and mass spectroscopy techniques to improve the reliability of CSF analysis, and deepen our understanding of the progression and pathophysiology of AD (1). As a multimodal longitudinal AD-targeted study, ADNI has provided a myriad of important insights on various aspects of AD, emphasizing AD pathophysiology and and disease progression.

S2.2 HCP

The original Human Connectome Project (HCP-Y) gathered data from 1,200 healthy young adults aged 21-35, including young adult sibships of average size 3–4, to build a high-quality data set that can be comparable with other populations. The primary goals of HCP include (i) building a "network map" that will shed light on the anatomical and functional connectivity within the healthy human brain, (ii) promoting the understanding of inter-individual variability of brain circuits to behavior, (iii) facilitating research into brain disorders, such as autism, AD, and schizophrenia, and (iv) making all data freely available to the scientific community (2; 3). Now, it has been extended to a number of studies on healthy humans ranging from birth to nonagenarians and beyond [†], aiming at mapping neural systems to underlying

[†]HCP lifespan, `https://www.humanconnectome.org/lifespan-studies/`

Downloaded from www.annualreviews.org. Guest (guest) IP: 152.19.89.75 On: Wed, 12 Mar 2025 14:59:29

cognition and behavior across the life span. Those studies will include HCP-B (HCP babies: age 0-5; 500 subjects), HCP-D (HCP development: age 5-21; 1,350 subjects), and HCP-A (HCP aging: age 36-100+; 1,200+ subjects). All HCP studies are hybrid cross-sectional and longitudinal cohorts, which recruited participants according to specific inclusion and exclusion criteria, such as the age range, birth weight, no major diagnosed diseases, and informed consent, with longitudinal follow-up observations for subsets of participants. Such recruitment method ensures that the samples reflect the racial/ethnic and socioeconomic diversity of the US Census. The HCP collected various imaging modalities including DWI, rsfMRI, tfMRI, T1- and T2-weighted sMRI, and MEG/EEG. Domains of cognition, emotion, motor function, and sensation were also collected, while different major factors relevant to brain development, aging, cognition and behavior were collected for different age phases. For example, HCP-A collected the vascular burden (e.g., obesity, hypertension, smoking), risk gene status (e.g., *APOE*), hormonal status, and lifestyle factors (e.g., depression, sleep patterns, social/community engagement, and adversity) (3). For HCP-Y participants, genotyping data are available across 2 million SNPs from 1142 study participants, while for HCP study participants outside of the HCP-Y cohort, samples will be assayed on several SNP regions of interest. Follow-up samples will also be collected for longitudinal assessment. Two major advantages of HCP include maximized resolution of imaging data and overall data quality for multi-modal imaging.

S2.3 ABCD

The ABCD study is the largest prospective longitudinal study of brain development and child health in the United States, which has recruited approximately 11,880 children aged 9-10 years old from 21 research sites and is following them for 10 years into early adulthood [‡]. Its initial goal was to examine risk and resiliency factors associated with the development of

[‡]ABCD, `https://abcdstudy.org/about/`

Downloaded from www.annualreviews.org. Guest (guest) IP: 152.19.89.75 On: Wed, 12 Mar 2025 14:59:29

substance use, and then expanded far beyond, into identifying the underlying biospecimens, neural alterations, and environmental factors, and their contributions to the development of behavior, brain function, and other mental and physical outcomes throughout adolescence (4). The ABCD adopted multi-stage probability sampling strategy to recruit eligible children to reflect as best as possible, the sociodemographic variation of the US population. However, more neuroimaging research centers were located in urban areas, leading to a potential under-representation of rural youth. The ABCD study covers personal information, family structure, family socioeconomic status, medical history, mental/behavioral performance, lifestyle (physical activity, sleep, diet), substance use (both self-reported and screening: alcohol, nicotine, cannabis, caffeine, cocaine, and marijuana), exposure (air pollution and lead), neuroimaging data (sMRI, DWI, and rsfMRI and tfMRI), and genotyping data. At baseline and year 1 follow-up sessions, biological breath, saliva, urine, and hair samples were collected from youth and genotyping were performed from saliva and blood DNA sample for 11,601 participants. ABCD provides a comprehensive platform for investigating gene-environmental effects on children brain development.

S2.4 UKB

UKB is a very large prospective cohort study that have recruited over 500,000 individuals aged 40 and 69 from 22 centers across the United Kingdom. It aims to inspire the imaginations of health researchers around the world to meet the challenge of greater understanding, prevention, and treatment of a range of serious illnesses [§]. Extensive phenotypic and genotypic details about its participants were collected, including data from questionnaires focused on health and lifestyle, physical measures, sample assays, accelerometry, multimodal imaging, genome-wide genotyping and longitudinal follow-up for a wide range of health-related outcomes. The UKB imaging study is by far the largest multi-modal imaging study in the

[§]UK Biobank, `https://www.ukbiobank.ac.uk/`

world, with over 50,000 participants having undergone assessments (5), including brain sMRI, brain fMRI, brain DWI, body MRI, low-dose X-ray bone and joint scans, and ultrasound of the carotid arteries. The genotype data, whole exome sequencing, and whole genome sequencing data for 500,000, 470,000, and 200,000 participants are available to researchers up till 2022, respectively. Finally, over 19,155 diagnostic terms has been collected including hospitalization episode statistics (HES) and recorded using the International Classification of Diseases, Tenth Revision (ICD-10) codes. There is expected to be 20 years of longitudinal follow-up on the participants, and the identification of disease risk factors should increase over time with emerging clinical outcomes. The UKB data set provides a unique opportunity for uncovering the genetic bases of brain structure and function, aging, and various diseases. For the recruitment procedure, postal invitations were sent to 9,238,453 individuals aged 40–69 years old, who lived within 25 miles from one of 22 assessment centres in the UK. With a response rate of 5.5%, there is significant evidence of selection biases, including the higher socio-economic status, better education and health of the UKB sample compared to the general population, leading to debates on the generalizability of UKB findings. Nevertheless, as reported in (6), many findings from UKB appear to be generalizable to England and Scotland.

S2.5 ENIGMA

The Enhancing NeuroImaging Genetics through Meta-Analysis (ENIGMA) Consortium is a global alliance of over 1,400 scientists across 43 countries in the fields of imaging genomics, neurology, and psychiatry, studying a range of large-scale human brain studies that integrate data based on sMRI, DWI, fMRI, genetic data and many patient populations from over 70 institutions worldwide (7). Launched in December 2009, the initial goal of the ENIGMA was to discover the impact of genetic factors on brain systems by integrating the two big data sources—neuroimaging and genetics. The major goals of ENIGMA

Downloaded from www.annualreviews.org. Guest (guest) IP: 152.19.89.75 On: Wed, 12 Mar 2025 14:59:29

include[¶] (i) pushing forward the field of imaging genetics, (ii) ensuring promising and reproducible findings, (iii) sharing data, ideas, methods, algorithms and other information, and (iv) training new investigators. The consortium consists of over 50 working groups (WGs), including diagnosis-based, normal variation-based, and method-based WGs. In 2014, ENIMGA considered nine targeted disorders: schizophrenia, bipolar disorder, major depressive disorder, obsessive-compulsive disorder, attention-deficit/hyperactivity disorder, autism spectrum disorders, substance use disorders, 22q11.2 deletion syndrome, and the effects of the human immunodeficiency virus on the brain. Following this, additional work groups focusing on specific disorders were established, including anxiety disorders, suicidal thoughts and behavior, sleep and insomnia, eating disorders, irritability, antisocial behavior, and dissociative identity disorder. Besides the diagnosis-based WGs, normal-variation WGs study the brain lifespan development, normal aging, gender difference, sleep patterns, and early onset psychosis, whereas method-based WGs span over developing innovative pipelines on producing DWI measures, anatomical shape measures, and data harmonization. Up till now, ENIGMA has stood out for its great impact in promoting robustness and reproducibility, setting methodological standards, and driving new discoveries in neuroscience research and clinical translation.

References

[1] Weiner MW, Veitch DP, Aisen PS, Beckett LA, Cairns NJ, et al. 2017. The alzheimer's disease neuroimaging initiative 3: Continued innovation for clinical trial improvement. *Alzheimer's & Dementia* 13(5):561–571

[2] Van Essen DC, Smith SM, Barch DM, Behrens TE, Yacoub E, et al. 2013. The WU-Minn human connectome project: an overview. *NeuroImage* 80:62–79

[¶] ENIGMA, `https://enigma.ini.usc.edu/`

Downloaded from www.annualreviews.org. Guest (guest) IP: 152.19.89.75 On: Wed, 12 Mar 2025 14:59:29

[3] Bookheimer SY, Salat DH, Terpstra M, Ances BM, Barch DM, et al. 2019. The lifespan human connectome project in aging: an overview. *NeuroImage* 185:335–348

[4] Karcher NR, Barch DM. 2021. The abcd study: understanding the development of risk for mental and physical health outcomes. *Neuropsychopharmacology* 46(1):131–142

[5] Littlejohns TJ, Holliday J, Gibson LM, Garratt S, Oesingmann N, et al. 2020. The uk biobank imaging enhancement of 100,000 participants: rationale, data collection, management and future directions. *Nature Communications* 11(1):1–12

[6] Batty GD, Gale CR, Kivimäki M, Deary IJ, Bell S. 2019. Generalisability of results from uk biobank: Comparison with a pooling of 18 cohort studies. *MedRxiv* :19004705

[7] Thompson PM, Jahanshad N, Ching CR, Salminen LE, Thomopoulos SI, et al. 2020. Enigma and global neuroscience: A decade of large-scale studies of the brain in health and disease across more than 40 countries. *Translational psychiatry* 10(1):1–28

Downloaded from www.annualreviews.org. Guest (guest) IP: 152.19.89.75 On: Wed, 12 Mar 2025 14:59:29
