# Supplementary material for: Statistical Learning Methods for Neuroimaging Data Analysis with Applications
Source: Annu Rev Biomed Data Sci. Author manuscript; Available in PMC 2025 Apr 2. (PMC11962820; doi:10.1146/annurev-biodatasci-020722-100353)
Supplement: Supplementary Tables [file NIHMS2065436-supplement-Supplementary_Tables.pdf]

Supplemental Material: Annu. Rev. Biomed. Data Sci. 2023. 6:73-104
https://doi.org/10.1146/annurev-biodatasci-020722-100353

# Supplementary Tables for "Statistical Learning Methods for Neuroimaging Data Analysis with Applications"

Downloaded from www.annualreviews.org. Guest (guest) IP: 152.19.89.75 On: Wed, 12 Mar 2025 14:59:29

Hongtu Zhu[1], Tengfei Li[2], and Bingxin Zhao[3]

[1]Departments of Biostatistics, Statistics, Genetics, and Computer Science and Biomedical Research Imaging Center, University of North Carolina, Chapel Hill
[2]Departments of Radiology and Biomedical Research Imaging Center, University of North Carolina, Chapel Hill
[3]Department of Statistics and Data Science, University of Pennsylvania

Table S1: Summary of key information for eight neuroimaging modalities

| Modality | Tracer | Resolution | Feature | Use | Software |
|---|---|---|---|---|---|
| sMRI (T1, T2) | Fluid characteristics of different tissues | 0.5-1 mm | Cortical thickness, cortical folding, sulcal depth, voxel-based morphometry, regional volumes and shape | Measure brain cortical/subcortical structural changes for diagnosis/staging/follow-up of disease/brain development. | Freesurfer, ANTs, FSL, SPM, AFNI, Hammer, BRAIN-Visa, BrainSuite |
| DWI | Brownian motion of water molecules within voxels | 1.25-3 mm | Fractional anisotropy, axial/radial/mean diffusivity, DKI/ NODDI parameters, structural connectivity | Delineate tumors, suspected acute ischemic brain injury, intracranial infections, masses, trauma, and edema; map structural connectome in research. | FSL, Mrtrix, AFNI, TrackVis, Camino, TORTOISE, slicerDMRI, Dipy, CAMINO, DSIStudio |
| fMRI | Blood-oxygen-level-dependent (BOLD) response in blood flow associated with brain function | 3-4 mm (spatial); 1-3 s (temporal) | Beta image, functional connectivity, weighted and binary network metrics | Brain activity mapping under tasks, brain abnormalities detection, pre-operative brain functional mapping. | SPM, FSL, AFNI, CPAC, FuNP |
| PET | Emissions from radioactive tracers | 4-5 mm | Standard uptake ratio | Reveal metabolic/ biochemical functions of tissues/organs and abnormalities in brain neurophysiology/ neurochemistry | NiftyPET, SPM, Metavol, NEUROSTAT, APPIAN, kinfitr, LIFEx, Pypes, SPAMALIZE |
| CT | X-ray attenuations by different tissues inside the body | Tens of nanometres-5 mm | Local and regional volumetric/thickness measures, tumor features | Diagnosing a range of conditions: abnormal blood vessels, brain atrophy, hemorrhage, swelling, stroke, tumors | ITK, SPM, PACS, Velocity, scenium, LIFEx |
| EEG | Electrical field produced by neuron electrical activity | 7-10 mm | Event-related potentials, connectivity/network measures, spectral content | Diagnosis and treatment of brain tumors, damage, dysfunction and disorders | EEGLAB, MNE, ELAN, FieldTrip, NUTMEG, Brain-Voyager, SPM |
| MEG | Magnetic field produced by neuron electrical activity, including tangential components of postsynaptic intracellular currents | 2-3 mm | Similar derived measures with EEG | Identification of brain functional areas (centers of sensory, motor, language and memory activities), precise location mapping of the source of epileptic seizures | EEGLAB, MNE, ELAN, FieldTrip, NUTMEG, Brain-Voyager, SPM |
| fNIRS | Changes in cortical BOLD response associated with brain function | 650-900 nm (spatial); milliseconds (temporal) | Similar derived measures with EEG and fMRI | Study normal and pathological brain physiology in infants/children | Homer2, Homer3, FNIRSOFT, OPENFNIRS, ICNNA, nirsLAB |

Downloaded from www.annualreviews.org. Guest (guest) IP: 152.19.89.75 On: Wed, 12 Mar 2025 14:59:29
